# Minimally expanded breast cancer tumor-infiltrating-lymphocytes provide guidance for therapeutic selection

**DOI:** 10.3389/fimmu.2025.1699262

**Published:** 2025-11-26

**Authors:** Andrea Aran, Gonzalo Lázaro, Vicente Marco, Vicente Peg, Maitane Faus, Laia Garrigós, José Pérez-García, Javier Cortés, Mercè Martí

**Affiliations:** 1 Immunology Unit, Department of Cell Biology, Physiology and Immunology, Institut de Biotecnologia i Biomedicina, Universitat Autònoma de Barcelona, Bellaterra, Spain; 2 Pathology, Hospital Quironsalud Barcelona, Barcelona, Spain; 3 Pathology Department, Vall d’Hebron University Hospital, Barcelona, Spain; 4 Department of Morphological Sciences, Universitat Autònoma de Barcelona, Bellaterra, Spain; 5 International Breast Cancer Center (IBCC), Pangaea Oncology, Quironsalud Group, Barcelona, Spain; 6 Scientific Department, Medica Scientia Innovation Research (MEDSIR), Barcelona, Spain; 7 Scientific Department, Medica Scientia Innovation Research (MEDSIR), Ridgewood, New Jersey, United States; 8 Department of Medicine, Faculty of Biomedical and Health Sciences, Universidad Europea de Madrid, Madrid, Spain; 9 IOB Madrid Institute of Oncology, Hospital Beata María Ana, Madrid, Spain; 10 Biosensing and Bioanalysis Group, Institut de Biotecnologia i Biomedicina, Universitat Autònoma de Barcelona, Bellaterra, Spain

**Keywords:** breast cancer, TIL, tumor-infiltrating lymphocytes, immunotherapy, TCR repertoire

## Abstract

**Introduction:**

The analysis of tumor-infiltrating lymphocytes often requires techniques that expand their numbers, potentially introducing bias. To address this, we performed a detailed analysis of minimally cultured TILs to evaluate whether this approach better preserves their characteristics.

**Methods:**

The TIL culture method was based solely on tumor tissue with low IL-2 supplementation to minimize artificial alterations. The validity of this approach was confirmed by the correlation between CD3+ T cell percentages in cultures and infiltration patterns observed by immunohistochemistry. Immunophenotyping, cytokine release, and TCR repertoire analysis were used to characterize CD4+ and CD8+ T cell subsets and their molecular features during minimal expansions.

**Results:**

High TIL infiltration areas did not consistently correspond to an increased presence of any T cell subset; both CD4+ and CD8+ T cells frequently coexisted in these regions. In contrast, low TIL infiltration sections often displayed a higher proportion of CD4+ T cells. An inverse correlation between CD4+ T cell percentages and cytotoxic molecules was observed, indicating reduced cytotoxic activity in low-TIL sections with abundant CD4+ T cells. TCR repertoire analysis revealed differences between T cell subsets: CD4+ T cells were associated with longer TRA CDR3 nt and shorter TRB N(D)N nt lengths, along with lower diversity, while CD8+ T cells did not exhibit significant correlation with any TCR feature.

**Discussion:**

This study highlights the distinct biological features of CD4⁺ and CD8⁺ TIL populations within the tumor microenvironment that can be preserved using a minimally expanded TIL approach. The observed associations between IHC patterns, T cell subset composition, cytotoxic potential, and TCR repertoire diversity help identify which biopsy regions yield TILs with greater therapeutic potential, thus providing guidance for TIL selection in immunotherapy.

## 1 Introduction

Breast cancer (BC) is the most common cancer in women and the most prevalent worldwide according to the World Health Organization (WHO). In 2022, 2.3 million women were diagnosed with BC and it has a growing incidence (1, 2). In the same year, about 670,000 women died from the disease (1). The early diagnosis is a crucial factor in survival, as tumors in early stages, that is, without disease beyond the breast and regional lymph nodes, have a high probability of cure, with an overall survival rate of about 80% (3). However, metastatic BC are treatable but rarely curable and most of the available treatments aim to extend survival years and to improve patients’ life quality (3).

The antitumor immunotherapy aims to restore, enhance, or modulate the patients’ immune system against tumors. In BC, it has shown a limited benefit but has a high potential and is currently being evaluated in multiple clinical trials in different disease contexts. Among the various types of immunotherapies, adoptive cell transfer (ACT) of tumor-infiltrating lymphocytes (TILs) has demonstrated remarkable success in combating certain types of cancer. TILs have demonstrated a predictive and prognostic value for neoadjuvancy (4), adjuvancy (5, 6) and metastasis (7) in triple negative (TN) and HER2+ BC, highlighting the crucial role of T cells in the antitumoral response. Proof of its value is that TIL infiltration study in breast tumors using immunohistochemistry (IHC) is routinely performed during diagnosis (8), indicating that TILs play a protective role against breast tumors growth but also that BC is susceptible to treatment through TIL ACT. Some successful studies have been conducted in patients with metastatic BC through autologous TIL transfer (9), as well as with natural killer T cells (NKT) transfer (10). However, the mere presence of TILs within tumors does not guarantee a favorable clinical outcome and ACT success is still frequently constrained to certain patients and specific tumor types, which underscores the complexity of the antitumoral response.

One of the contributing factors to the variable success of ACT is the reliance on the expansion of TILs *in vitro* before their therapeutic application. This traditional approach has yielded positive results but may not be optimal in all cases. Recent research emphasizes the importance of *ex vivo* T cells or the so-called young TILs as an alternative approach for ACT (11). These T cells may exhibit distinct properties over the *in vitro* expanded counterparts, including their phenotypic attributes, antigen reactivity, and metabolic profiles. Understanding these distinctions can lead to the development of more efficient and clinically relevant treatments, but the limited number of cells that can be extracted from a biopsy also limits the ability to perform various phenotypic and functional studies. Thus, to better comprehend the natural status of TILs within tumors, it is essential to investigate the composition of *ex vivo* and/or these minimally cultured TILs, along with their activation status, specificity, and functionality. This comprehensive analysis should encompass factors associated with their function and their contribution to the antitumoral response.

As CD4+ and CD8+ T cells are associated with the release of certain cytokines, they can be used as a marker of function within the tumor infiltrate. Cytokine patterns secreted by different CD4+ T cell subtypes (Th1, Th2, Th17, Tfh, and Treg) (12) are directly related with their functions. Likewise, CD8+ T cells, or cytotoxic T lymphocytes (CTLs) execute the lysis of target cells by releasing cytotoxic factors (perforins, and granzymes and granulysin) or by the interaction of the of the FasL with Fas, inducing apoptosis of tumor cells.

On the other hand, the T cell receptor (TCR), which provides antigen specificity to T cells, serves as a marker for monitoring the status and evolution of TILs. Diversity analysis reflects the number of distinct clones and their abundance, allowing for the identification of clonal expansions, indicating T cell activation and response. Additionally, we have previously analyzed the TCR repertoire in expanded CD4+ and CD8+ TILs reporting certain differences in their TCRs, some of them intrinsic to T cell subsets but others associated with the antitumoral response (13). However, the original repertoire may be biased during TILs expansions due to the greater proliferative capacity of certain clones. This has recently been demonstrated, although it was also observed that the expansion of TILs from different sections of the same tumor resulted in highly divergent repertoires (14). Consequently, culturing small sections of the same biopsy allows for multiple samples of TILs from the same tumor, but with different repertoires, encompassing a larger part of the original sample. Considering all this, the aim of this study was to explore these characteristics in TILs derived from 11 BC biopsies. Sections of each biopsy were cut and separately cultured as explants with no reagents used other than an external low supply of IL-2 (100U/ml) with the aim of preserving the originally divergent repertoires as much as possible (14).

First, we aimed to correlate these findings with the degree of TIL infiltration observed in the initial IHC analysis. This approach was motivated by the need to bridge the gap created when TILs from biopsies are cultured by disaggregating tumors, which disrupts the spatial organization of lymphocytes within the native microenvironment. By culturing multiple small explants that were direct “mirror” counterparts of the IHC-analyzed tissue, we sought to understand how the initial composition of TILs within the tumor microenvironment correlates with their behavior upon culture. By stratifying our analysis based on IHC results indicating high or low TIL infiltration, we sought to elucidate whether differences in the early explant culture values, such as percentages of CD3+ T cells, CD4+, CD8+ T cell subsets, CD8/CD4 ratio, cytokine production and TCR repertoire features, could be associated with the degree of TIL infiltration observed in the tumor tissue. A correlation study has been conducted between the phenotype of TILs and the other studied properties, with the goal of identifying which of them can provide more information about the phenotypic and functional characteristics of the original tumor infiltrate. This comprehensive approach provides valuable insights into the functional and phenotypic characteristics of TILs within the tumor microenvironment and enhances our understanding of their potential role in antitumoral immune responses.

## 2 Materials and methods

### 2.1 Breast cancer biopsies

Four triple-negative (TN), three luminal A (LA), and four luminal B (LB) breast cancer biopsies were obtained from surplus hospital material donated by Hospital Quirón of Barcelona and Hospital Vall d’Hebron using standard surgical procedures, with the appropriate approval of the Ethical and Scientific Committee of the institutions. Consent was obtained from patients according to the local institutional review board requirements. Biopsies were cut into small slices and cultured as explants in 48-well plates, placing one portion per well, covering different parts from the entire tissue sample to maintain representation of TILs at different locations in the surgical sample, summarized in **Table 1**. In five of the biopsies with sufficient material, samples were first cut into two mirror-image portions: one used for IHC to determine TIL distribution, and the other used for *in vitro* analyses.

**Table 1:** Summary of breast cancer biopsies and corresponding sections used in the study.

Breast cancer type	Biopsy	Age	Infiltration degree^a^	Section	Infiltration degree of section
TN	BC-PS-562 (562)	44	High	562.2	na
				562.4	na
				562.6	na
				562.7	na
				562.9	na
				562.10	na
				562.11	na
TN	BTLQ1 (Q1)	58	Inter	Q1.1B	na
				Q1.3D	High
				Q1.3B	Low
				Q1.4D	High
				Q1.4B	Low
				Q1.6D	High
				Q1.6B	Low
				Q1.8D	High
				Q1.9D	High
TN	BTLQ2 (Q2)	61	Inter	Q2.2D	Low
				Q2.2M	Low
				Q2.4D	Low
				Q2.4M	Low
				Q2.4B	High
				Q2.6D	Low
				Q2.6B	High
				Q2.8D	Low
				Q2.8B	High
				Q2.9B	High
LB	BTLQ7 (Q7)	43	High	Q7.5	na
				Q7.6	na
				Q7.7	na
				Q7.8	na
LB	BTLQ8 (Q8)	42	Low	Q8.3	High
				Q8.4	High
				Q8.5	High
TN	BTLQ10 (Q10)	63	Inter	Q10.1	Low
				Q10.3	High
				Q10.5	Low
LB	BTLQ12 (Q12)	55	Inter	Q12.1	na
				Q12.2	na
				Q12.3	na
				Q12.4	na
				Q12.5	na
LA	BTLQ14 (Q14)	48	Inter	Q14.2.1	na
				Q14.2.2	na
				Q14.2.3	na
				Q14.2.4	na
LA	BTLQ15 (Q15)	55	Low	Q15.2.1	High
				Q15.2.2	Low
				Q15.2.3	High
LA	BTLQ16 (Q16)	63	Inter	Q16.1.1	na
				Q16.2.1	na
LB	BTLQ17 (Q17)	74	Low	Q17.3.1	na
				Q17.3.2	na
				Q17.3.3	na
				Q17.3.5	na

Information includes breast cancer type, patient age, overall tumor infiltration degree, specific section identifiers, and the infiltration degree of each section. Cells highlighted in grey indicate samples used for TCR sequencing. ^a^Infiltration defree defined following the criteria stablished by Salgado et al. (8). Na, not achieved.

### 2.2 Immunohistochemistry

The IHC of biopsies was performed by Pathological Anatomy Unit of Hospitals. The presence of tumoral cells and infiltrating T cells in the biopsies was determined by hematoxylin and eosin (HE) and IHC using an anti-human CD3 (clone 2GV6), respectively.

### 2.3 T cell cultures

Biopsy-derived T cells were cultured in 1 ml of complete T cell culture medium [IMDM GlutaMAX™ (Gibco) + 10% decomplemented human serum + antibiotic/anti-mycotic (Sigma)], where 100U/ml of IL-2 (provided by the NIH) was added. Half of the medium with the corresponding IL-2 was renewed every 5 days, while the removed portion was frozen for later cytokine analysis. Early explant cultures were maintained between 2 and 4 months, depending on the biopsy, in incubators at 37°C and 5% CO2.

### 2.4 Flow cytometry

Between 2–5 x 10^5^ cells were stained with anti-human antibodies for 20 min at 4°C in the dark in PBS + 2% fetal bovine serum (FBS). After incubation, cells were washed and analyzed by flow cytometry. Antibodies used for flow cytometry were FITC-conjugated anti-human CD3 (RRID: AB_10893003), PE-conjugated anti-human CD4 (RRID: AB_393790), and APC-conjugated anti-human CD8 (RRID: AB_10642579) (BD Pharmingen). A BD FACS Canto flow cytometer was used for analysis. Analyses were performed using the FlowJo v10.4, (RRID: SCR_008520), and FACS Diva software. For each sample, lymphocytes were gated using FSC/SSC parameters, followed by CD3+ identification and subsequent discrimination of CD4+ and CD8+ subsets. Percentages of these subsets were calculated within the CD3+ compartment.

### 2.5 Cytokine analysis

For the analysis and quantification of soluble molecules, supernatants collected on days 5, 10, 15, and 20 from the early explant T-TIL cultures were used. The secretion of 11 human molecules was studied, including IL-4, IL-10, IL-17A, IFN-γ, TNF-α, soluble Fas (sFas), soluble Fas ligand (sFasL), granzyme A, granzyme B, perforin, and granulysin, using the LEGENDplex™ Human CD8/NK Panel (13-plex) bead-based kit (BioLegend) and following the manufacturer’s instructions. Analyses were performed using BD FACSCanto and CytoFlex S (Beckman Coulter Life Sciences) equipment.

### 2.6 TCR library preparation

Between 1 × 10^5^ - 1 × 10^6^ cells from the early explant cultures were collected after 7–15 days and used for RNA extraction, isolated using the RNeasy Micro Kit (Qiagen) with on-column DNase digestion using an RNase-free DNase set (Qiagen) following the manufacturer’s instructions. The amount and integrity of RNA were measured externally by the Genomics Core Facility of the University Pompeu Fabra (UPF) using an Agilent 2100 Bioanalyzer (Agilent Technologies) and RNA 6000 Nano or RNA 6000 Pico chips. Samples with RIN <7 were excluded. TCR profiling was performed using a SMARTer Human TCR a/b Profiling Kit (Takara Bio, Shiga, Japan) and both TCR alpha and TCR beta chains were sequenced. Library purification was performed using Agencourt AMPure XP Beads (Beckman Coulter) and libraries were analyzed and validated on an Agilent 2100 Bioanalyzer using a DNA 1000 kit (Agilent Technologies). High-throughput sequencing (HTS) was performed on an Illumina MiSeq sequencer using a 600-cycle MiSeq Reagent Kit with paired-end 2 × 300 bp reads.

### 2.7 TCR repertoire analysis

Raw TCR sequencing data were aligned using MiXCR Immune Repertoire Analyzer (15) (RRID: SCR_018725) and processed using Immunarch (16) and VDJTools (17). Non-productive clonotypes were excluded and routine decontamination was performed to eliminate cross-sample contamination. Rarefaction curves were generated using Immunarch (16) to estimate the number of CDR3 nucleotide (CDR3nt) sequences expected when extrapolated to a defined sample size, corresponding to that of the largest sample. Before the length, biochemical properties and motifs analysis, pre-processed data were collapsed by CDR3aa sequences using VDJTools (17), that is, clonotypes with different nucleotide sequences encoding the same CDR3aa sequence were summed, and frequencies were recalculated. The length, biochemical properties and diversity were also analyzed using VDJTools (2) functions.

### 2.8 Statistical analysis and figures

Statistical analyses and figures were generated using GraphPad Prism, (RRID: SCR_002798) (version 7.0). The results of the statistical tests are shown in the figures.

## 3 Results

### 3.1 The percentage of CD3+ TILs in early explant cultures reflects IHC-based high versus low infiltration but is not associated with specific CD4+ or CD8+ subpopulations

The biopsies for TILs culture were cut into small explants covering the entire tissue sample, allowing for the preservation of TILs present in different locations within the obtained surgical biopsy (summarized in **Table 1**). Cells from the early explant cultures were collected around day 10 of culture when the number of lymphocytes allowed to perform characterization studies. The presence of CD4+ and CD8+ TIL subpopulations was studied by flow cytometry by analyzing the expression of CD3, CD4, and CD8 over the course of TIL culture (**Supplementary Figure 1**).

**Figure 1:**
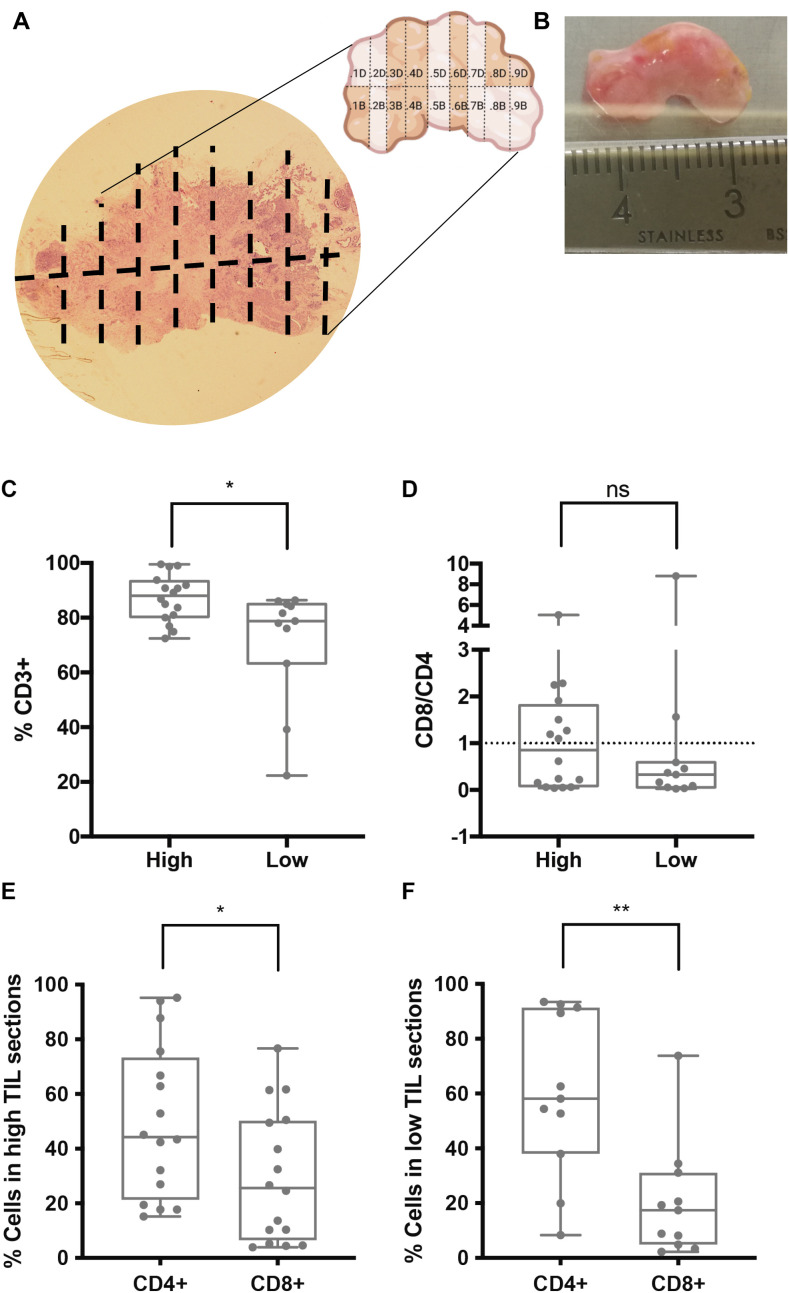
Association between IHC TIL infiltration and TIL populations in early explant cultures of tumor biopsies. **(A, B)** Representative biopsy (Q1) analyzed by IHC for CD3 to determine TIL infiltration. The IHC image **(A)** corresponds to the mirror of the biopsy, which was sectioned into multiple parts, some of which were cultured as early explants. The physical tumor tissue sample is shown in **(B)**. IHC results were used to categorize cultured sections as “high” or “low” TIL infiltration. **(C–F)** Percentages of T cell populations measured by flow cytometry in early explant cultures derived from sections classified as “high” or “low” TILs. **(C)** CD3+ T cells were significantly higher in cultures from “high TILs” sections (*p < 0.05). **(D)** CD8/CD4 ratio, **(E, F)** a significantly higher CD4+ versus CD8+ percentage was observed in both high **(E)** and low **(F)** TIL sections (*p< 0.05 and **p< 0.01, respectively). ns, not significant.

To assess the degree of TIL infiltration of each section, a “mirror” section was made from five biopsies for IHC analysis. This allowed for a direct comparison between the IHC results and the cultured explant sections (**Figures 1A, B**). The association between the IHC results and the biopsy sections enabled the categorization of the cultured sections into “high” or “low” TIL infiltration (8) in five of the 11 analyzed biopsies, where this comparison was possible (Q1, Q2, Q8, Q10 and Q15 biopsies) (**Table 1**). Based on this classification, the percentages of CD3+, CD4+, CD8+, and the CD8/CD4 ratio were examined in the different sections classified as high or low TIL.

We observed that sections classified as high TIL had a significantly higher percentage of CD3+ T cells compared to the low TIL sections (**Figure 1C**). Regarding the CD8/CD4 ratio, no significant differences were found between the two groups (**Figure 1D**). However, both high- and low-TIL sections showed a predominance of CD4+ T cells, a difference that was more pronounced in the low-TIL sections (**Figures 1E, F**), mainly due to a consistently lower percentage of CD8+ T cells in this group. These results suggested that CD4+, but not CD8+ T cells, tend to persist in early explant cultures but specially in those derived from low TIL sections.

Following the analysis of the association between IHC-defined TIL infiltration and early explant cultures, we next investigated the dynamics of T cell populations over the course of the cultures (**Supplementary Figure 1**). The composition of the biopsies was not homogeneous, and the percentages of TIL subtypes varied depending on the location of the section within the tumor. The CD3+ TILs population included CD4+, CD8+, double positive (CD4+CD8+, DP), and double negative (CD4-CD8-, DN) subtypes. CD8/CD4 ratios were calculated to facilitate comparison between these subpopulations. While certain sections did exhibit variations in the percentages of T cell populations over the culture period, a consistent pattern of predominance emerged among specific subpopulations. Notably, DP and DN T cells were also present in some biopsies; however, these subsets experienced the most pronounced changes throughout the culture duration (data not shown).

**Figure 2:**
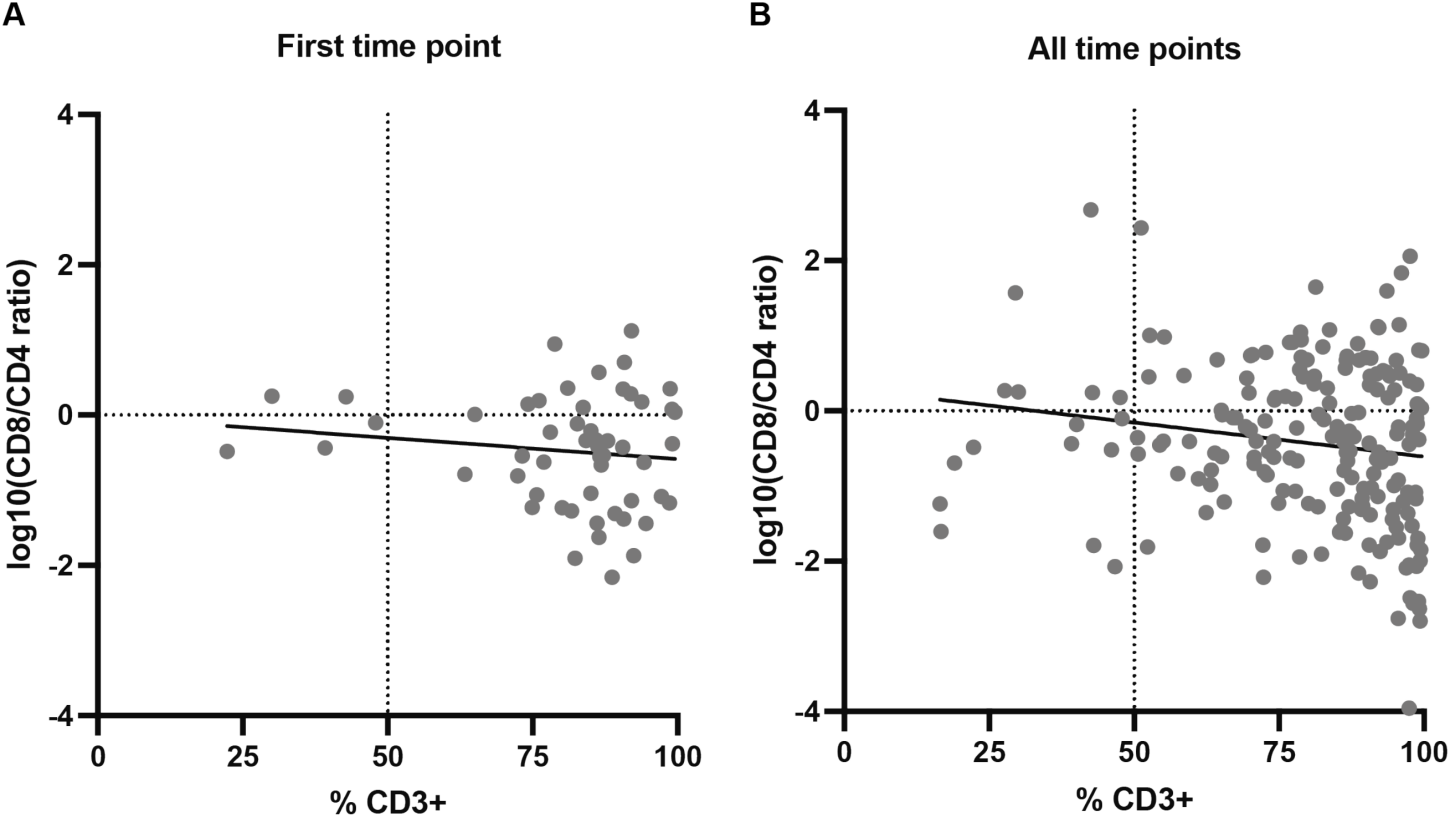
Correlation between percentages of CD3+ T cells and log10-transformed CD8/CD4 ratios in early explant cultures. **(A)** First timepoint of each explant culture (N = 53); no significant correlation was observed (Spearman r = -0.06, p-value (two-tailed) = 0.672), although a slight trend toward lower CD8/CD4 ratios with increasing CD3+ percentages is noted. **(B)** All timepoints combined (N = 192); a weak but significant negative correlation was detected (Spearman r = -0.169, p-value (two-tailed) = 0.019). Individual values for each biopsy-fragment and timepoint are shown in **Supplementary Figure 1**.

The correlation between the percentages of CD3+ T cells and CD8/CD4 ratios were calculated using flow cytometry data. CD8/CD4 ratios were log10-transformed for a better representation. We first compared data from the first time point, i.e., the first flow cytometry staining obtained from each explant (**Supplementary Figure 1**). Most sections had CD3+ T cell percentages above 60% and log10(CD8/CD4) values below 0, indicating higher percentages of CD4+ T cells than CD8+ T cells (**Figure 2A**). No significant correlation was observed, although a slight trend toward lower CD8/CD4 ratios with increasing CD3+ percentages can be appreciated. When considering all timepoints, which allowed us to determine if maintaining the early explant culture influenced the abundance of different subpopulations, most cultures still contained a high percentage of CD3+ T cells (**Figure 2B**), indicating that T cells can be maintained in the presence of the explant with low doses of IL-2 without any other reagent. A weak but significant negative correlation was detected, indicating that higher percentages of CD3+ T cells correlated with lower CD8/CD4 ratios in cultures, consistent with the tendency observed when analyzing first time points. This result could be explained by the inherently more proliferative nature of CD4+ T cells, causing them to remain abundant (**Supplementary Figure 1**). This is reinforced by the fact that this correlation was not observed in most of the early explant cultures nor in the IHC-flow cytometry association.

### 3.2 The percentage of CD4+ TILs in early explant cultures is inversely related to the presence of secreted molecules with cytotoxic functions, especially under low infiltration levels

To study the soluble molecules secreted by TILs, the supernatants from the early explant cultures of the biopsies were collected on days 5, 10, 15, and 20. Levels of IL-4, IL-10, IL-17A, IFN-γ, TNF-α, sFas, sFasL, granulysin, granzyme A, granzyme B, and perforin were measured using a bead-based flow cytometry assay. Most of the secreted cytokines were detected starting from days 10 and 15 (**Supplementary Table 1**). For each timepoint, cytokine concentrations (pg/mL) were correlated with the percentages of CD3+, CD4+, and CD8+ T cells measured in the corresponding culture on same timepoints, provided that the cytokine was detectable. Considerable heterogeneity was observed among sections from the same biopsies, and no specific cytokine pattern was shared across biopsies or sections.

**Figure 3:**
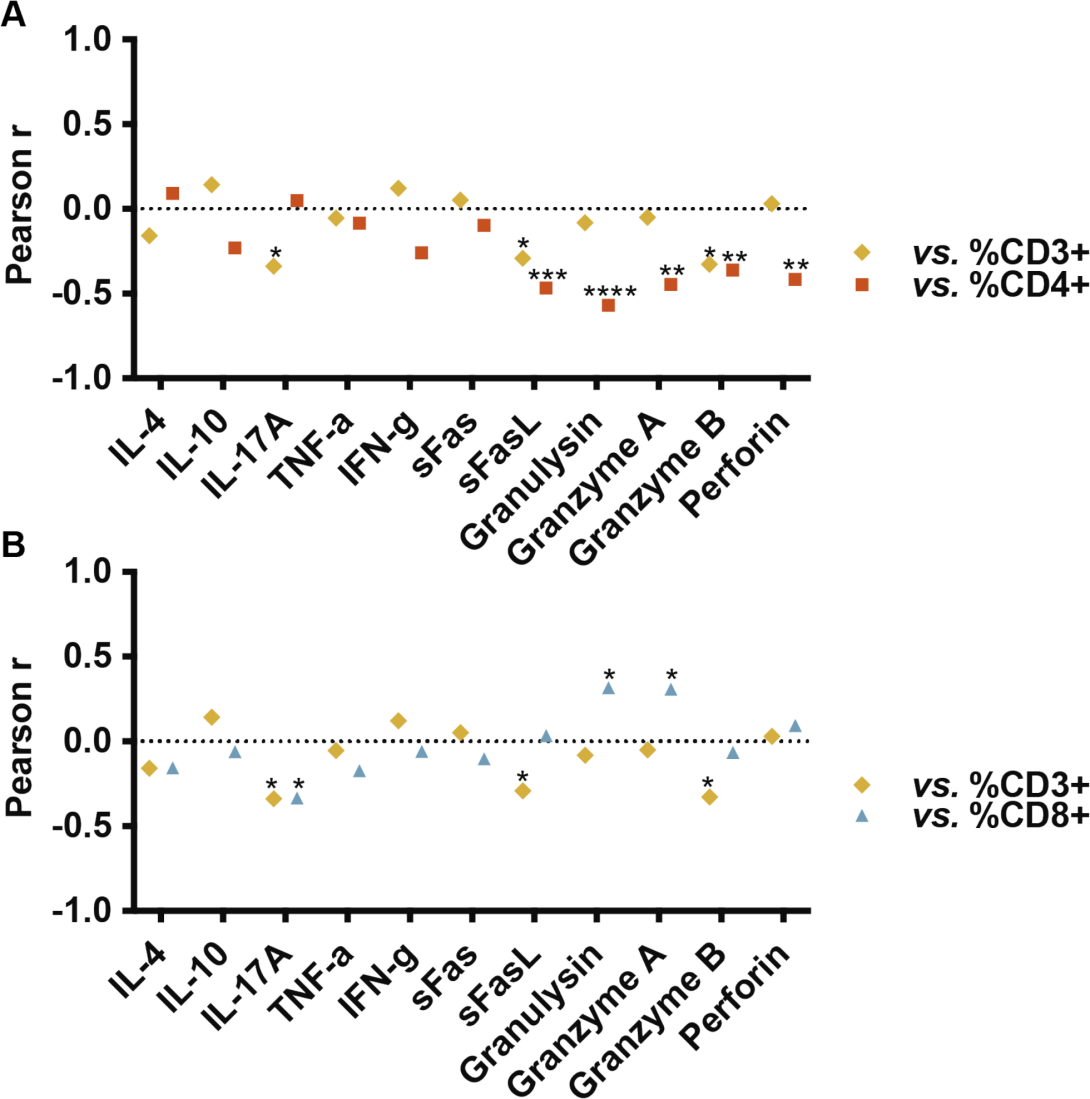
Pearson correlations between percentages of CD3+, CD4+ and CD8+ TILs and concentration (pg/ml) of soluble molecules in culture supernatants. CD4+ T cell percentages inversely correlated with certain cytotoxic molecules (sFasL, granulysin, granzyme A, granzyme B, and perforin; p = 0.0006, p < 0.0001, p = 0.0012, p = 0.0098, and p = 0.0026, respectively). CD3+ TILs also exhibited an inverse correlation with sFasL (p = 0.039) and granzyme B (p = 0.02). CD8+ percentages positively correlated with granulysin and granzyme A (p = 0.025 and p = 0.029, respectively). A significant inverse relationship with the production of IL-17A was observed both with the percentages of CD3+ (p = 0.017) and CD8+ T cells (p = 0.018). Individual cytokine values and dotplots for each population are provided in **Supplementary Table 1** and **Supplementary Figures 2-5**.

The relationship between the different soluble proteins secreted and the percentages of CD3+, CD4+, and CD8+ subpopulations, as well as the CD8/CD4 ratios, was analyzed using the values obtained from the staining at the same time points as the supernatant collection (**Supplementary Figures 2-5**). Certain correlations were observed when examining the percentages of CD3+, CD4+, and CD8+ but not for the CD8/CD4 ratios. Therefore, we analyzed whether the Pearson correlation patterns of the cytokines with the percentage of CD3+ could be associated with one of the two different T cell subsets (**Figure 3**).

By comparing the Pearson correlations obtained (**Supplementary Figures 2-4**), we observed correlation profiles for CD4+ and CD3+ TILs nearly inversely related (**Figure 3A**), while the profile for CD3+ and CD8+ TILs, maintained a similar pattern (**Figure 3B**). No correlations were observed between cytokines defining Th response patterns (IFN-γ in Th1, IL-4 in Th2, IL-17 in Th17) and CD4+ TILs. However, a significant inverse correlation was observed between CD4+ TILs and certain cytotoxic molecules, specifically sFasL, granulysin, granzyme A, granzyme B, and perforin (p = 0.0006, p < 0.0001, p = 0.0012, p = 0.0098, and p = 0.0026, respectively. Among these, the percentage of CD3+ was also inversely correlated with sFasL (p = 0.039) and granzyme B (p = 0.02). The percentages of CD8+ T cells showed a direct correlation with granulysin (p = 0.025) and granzyme A (p = 0.029). We also observed a significant inverse relationship with the production of IL-17A both with the percentages of CD3+ (p = 0.017) and CD8+ T cells (p = 0.018).

Overall, the data indicate that the correlation profiles of cytokines secreted with the percentage of CD3+ are more similar to those of CD8 +. However, a microenvironment with a more cytotoxic profile is better correlated with a lower amount of CD4+ T cells than with a higher amount of CD8+ T cells. This suggests that a high T cell infiltrate may be composed of both populations, while the greater percentage of CD4+ T cells generally becomes more noticeable, in terms of cytokine release, when the infiltrate is low, although correlations with the CD8/CD4 ratio could not be appreciated (**Supplementary Figure 5**).

### 3.3 Characterization of the TCR repertoire of TILs in the early explant cultures

To characterize the intra-tumoral TCR repertoire, a preliminary descriptive analysis was performed on the early explant cultures. The distribution of clones based on their frequencies, the nucleotide (nt) size of CDR3 sequences (CDR3 nt length), the biochemical properties of CDR3 amino acid (CDR3 aa) sequences, and the diversity were analyzed for both TRA and TRB sequences.

We obtained a total of 39,812 TRA and 35,357 TRB nucleotide sequences from 10 out of 11 biopsies, from which a sufficient number of cells was available for sequencing (**Supplementary Table 2**). The richness (number of different clones or sequences) was analyzed in each biopsy based on its sample size (number of reads). Results were compared using rarefaction curves, which allow the extrapolation of the number of observed clones to the sample with the highest number of reads (in this case, the biopsy 562). A similar richness was observed in almost all biopsies, except for biopsy 562 in the TRA analysis and biopsy Q7 in both the TRA and TRB analysis (**Supplementary Figures 6A, B**). Considering the sample size, biopsy Q7 had a very high number of clonotypes (8,803 TRA nt sequences and 10,457 TRB nt sequences). The number of sequences per biopsy section was relatively similar within each biopsy, both in TRA and TRB sequences, except for biopsy 562, which showed highly variable values in the four sections studied (**Supplementary Figures 6C, D**).

The average CDR3 nt length was 42 nt and 44 nt for TRA and TRB sequences (**Supplementary Figure 7A**). The N(D)N nt region had an average size of 5 nt in TRA sequences and 13 nt in TRB sequences (**Supplementary Figure 7B**), which was expected due to the presence of the D region in TRB sequences missing in TRA sequences. The CDR3 nt length showed a normal distribution in all biopsies, both in TRA and TRB sequences (data not shown). Next, we analyzed the biochemical properties (hydrophobicity, charge, and polarity) of the central 5 aa of CDR3 aa sequences. The obtained values were weighted by sequence frequency and normalized by dividing amino acid property values by CDR3 central 5 sub-region size (17). Overall, the mean values obtained were similar between TRA and TRB sequences (**Supplementary Figure 7**). The mean hydrophobicity values were -0.99 and -0.72, and the mean polarity values were 0.56 and 0.51 for TRA and TRB sequences, respectively (**Supplementary Figures 7C, E**). The mean charge values were around 0 for both groups (**Supplementary Figure 7D**).

**Figure 4:**
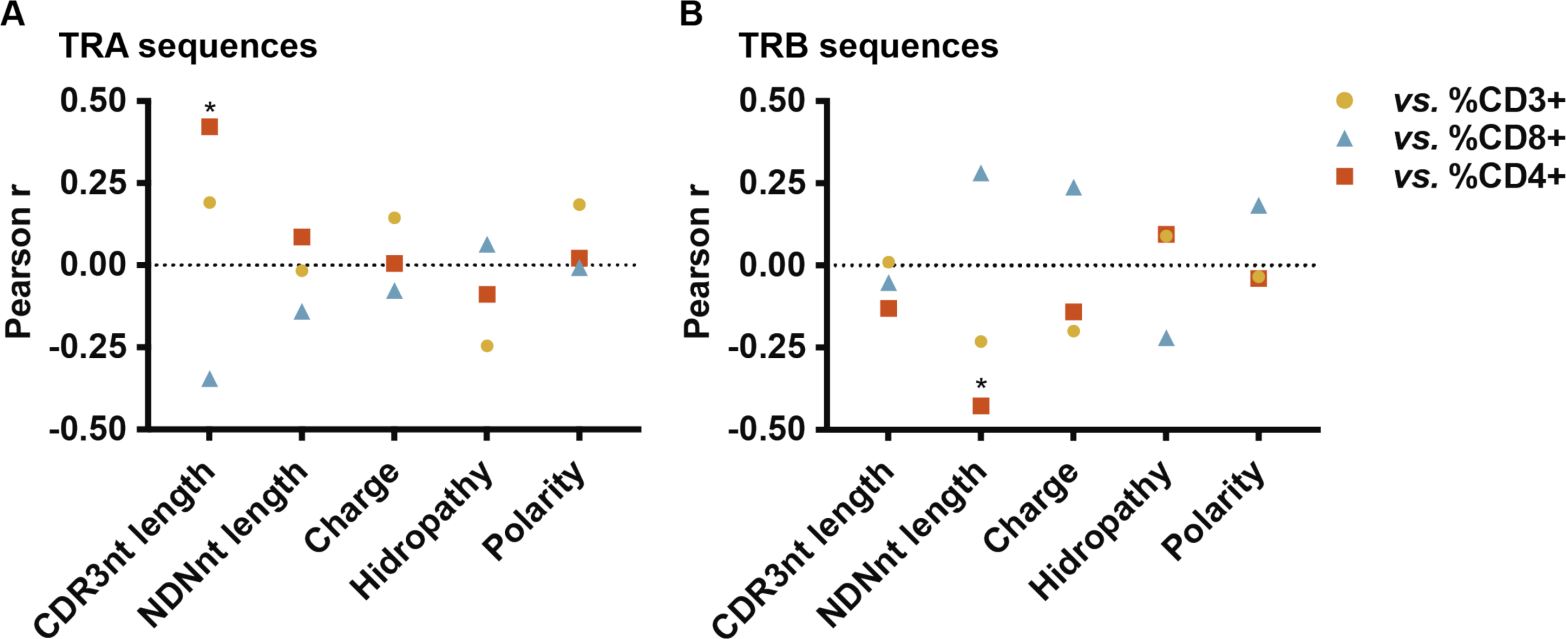
Pearson correlations between TCR sequence properties and CD3+, CD4+ and CD8+ percentages in minimally cultured TILs. **(A)** TRA CDR3 nt length positively correlated with CD4+ T cell percentage (p = 0.03). **(B)** TRB N(D)N nt length negatively correlated with CD4+ T cell percentage (p = 0.03). Individual data points are shown in **Supplementary Figure 7**.

We examined correlations between the percentages of CD3+, CD4+ and CD8+ T cells in minimally cultured TILs with the TCR features analyzed: CDR3 nt length, N(D)N nt length size, hydrophobicity, charge, and polarity of the central 5-mer region of CDR3 aa, for both TRA and TRB sequences (**Figure 4**). A significant positive correlation was observed between the percentage of CD4+ T cells and the CDR3 nt length in TRA sequences (p = 0.03) (**Figure 4A**). Additionally, an inverse correlation was found between CD4+ T cell percentages and the N(D)N nucleotide size in TRB sequences (p = 0.03) (**Figure 4B**), suggesting that CD4+ TILs exhibit longer CDR3 nucleotide sequences in TRA but shorter N(D)N nucleotide regions in TRB sequences. No correlations were found between the percentages of CD3+ or CD8+ T cells and the TCR properties. However, the correlation profile for CD3+ TILs was more similar to that of CD4+ TILs in this analysis, indicating that CD3+ cells may include a significant proportion of CD4+ TILs.

### 3.4 CD4+ TIL percentages in early explant cultures are inversely related to TCR repertoire diversity

In addition to examining the physicochemical CDR3 properties, the repertoire analysis measures provide insights into the immune response. One of the most used parameters is the diversity, which measures whether the infiltrating lymphocytes exhibit a broad range of TCRs or, in contrast, if there is a high clonality, indicating oligoclonal expansions. The diversity analysis of the TCR repertoire was performed using the normalized Shannon-Wiener index (nS-W), which combines richness and abundance. Diversity was examined separately for TRA and TRB sequences as a control, ensuring similar indices were obtained for both sequence groups within each sample (**Supplementary Table 2**). The diversity indices obtained for TRA and TRB sequences showed a significant correlation (p<0.0001), validating the data obtained from parallel sequencing (**Supplementary Figure 8A**). The mean diversity indices for TRA and TRB sequences were very similar, at 0.44 and 0.47, respectively (**Supplementary Figure 8B**). However, there was a high dispersion, indicating variable diversities among different samples. Except for certain biopsies such as Q8 or Q7 (which exhibited high diversity indices in all sections) most biopsies exhibited different diversity indices in the analyzed sections, demonstrating the heterogeneity of TIL within the same biopsy (**Supplementary Table 2**).

**Figure 5:**
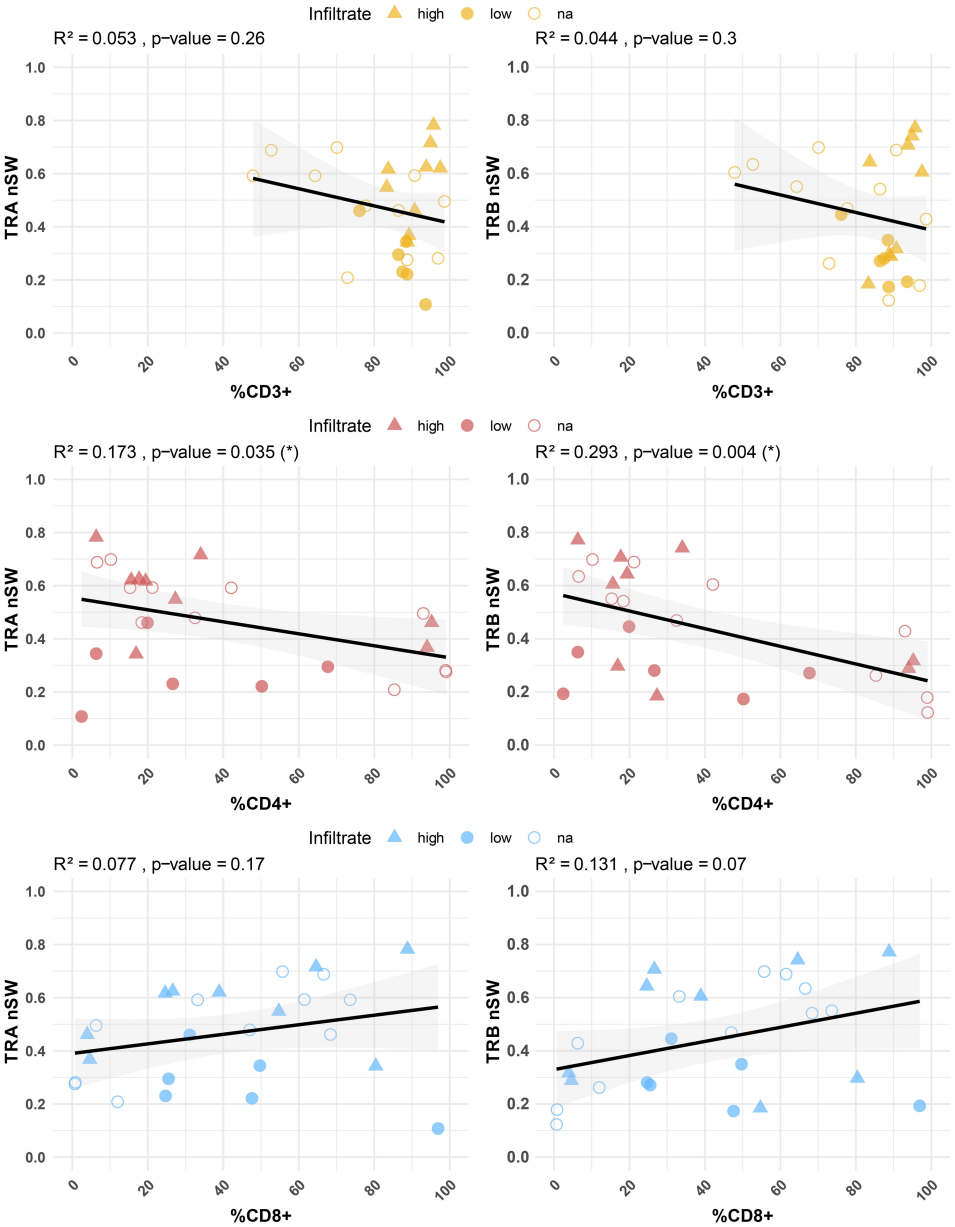
Correlation between TCR diversity (nS-W index) and percentages of CD3+, CD4+ and CD8+ T cells in minimally cultured TILs. Significant correlations were observed between CD4+ T cell percentages and diversity indices in both TRA (p = 0.03) and TRB (p = 0.004) sequences. IHC-defined infiltration levels are indicated: high (triangle) and low (filled circles). Unknown samples are represented by unfilled circles. Individual repertoire values are shown in **Supplementary Figure 8**.

Considering this dispersion and to investigate a possible relationship with TIL phenotype, the diversity indices were correlated with the percentages of CD3+, CD4+, and CD8+ TILs (**Figure 5**). A significant inverse correlation was found between the percentages of CD4+ T cells in the early explant cultures and the nS-W diversity index for both TRA and TRB sequences (p = 0.035 and p = 0.004, respectively). In contrast, the percentage of CD8+ T cells did not show a significant correlation with repertoire diversity, although there was a tendency towards greater diversity with higher CD8+ T cell percentages. Notably, the pattern observed for CD3+ T cell percentages was more similar to that of CD4+ T cells; specifically, diversity diminished with an increasing number of CD3+ T cells. When analyzing early explants cultures derived from high- versus low-TIL sections, we observed that CD4+ T cell diversity remained high in cultures derived from high-TIL sections even when the relative frequency of CD4+ T cells was lower, likely due to the concomitant abundance of CD8+ T cells, whereas in cultures derived from low-TIL sections, CD4+ diversity was consistently reduced regardless of CD4+ frequency. In contrast, for CD8+ T cells, early explant cultures derived from low-TIL sections also exhibited low diversity, while those derived from high-TIL tumors displayed a broader range. Overall, the data revealed that the percentages of CD4+ and CD8+ T cells in TIL cultures were associated with distinct characteristics of TCR phenotype and repertoire.

## 4 Discussion

In this study, we conducted an in-depth characterization of minimally cultured TILs in three different types of BC tumors (three LA, four LB and four TN), focusing on CD4+ and CD8+ T cells in primary cultures, the cytokine profile secreted in these cultures, and an analysis of the TCR repertoire. Although the patient cohort was small and included heterogeneous subtypes, our aim was not to correlate immune parameters with tumor type or clinical outcome. When considering the number of explant cultures analyzed rather than the number of patients, our dataset offers a reasonable basis to explore TIL behavior under minimally expanded conditions. Additionally, we employed IHC to determine whether the differences observed in the early explant cultures could be associated with the degree of TIL infiltration present in the tumor tissue. Our findings reveal insights into the heterogeneity of TIL populations, their behavior in culture, and their potential impact on the tumor microenvironment.

When stratified by IHC-defined levels of TIL-infiltration – high and low – a clear correlation emerged between the percentage of CD3+ T cells in early explant cultures and the IHC results. Specifically, tumors sections exhibiting high levels of infiltration as determined by IHC corresponded to a significantly elevated percentage of CD3+ T cells in culture, and vice versa. This finding is important as it demonstrates that minimally cultured TILs maintain the original level of infiltrate, resulting in less bias compared to other *in vitro* practices, such as rapid expansion methods that artificially boost TIL numbers.

When analyzing the percentages of CD4+ and CD8+ T cells, no significant associations were found between the high or low infiltration sections and either T cell subset. Notably, a higher percentage of CD8+ T cells was generally observed in sections with high infiltration, whereas this pattern did not apply to CD4+ T cells, which were present regardless of the overall infiltration level. This suggests that low infiltration levels may impede the maintenance of high CD8+ T cell percentages, likely due to their dependence on support from other cell types, including CD4+ T cells. In contrast, CD4+ T cells, probably due to their functional variability, can maintain high percentages independently. This dynamic, as reflected in our cultures, aligns with physiological processes.

Our first observation was the marked spatial heterogeneity of TIL distribution within the biopsies. Different sections of the same tumor exhibited different percentages of CD3+ T cells, which has been previously documented in breast tumors using IHC (18–20). This spatial heterogeneity was even more evident when analyzing T cells subsets, indicating that TILs subsets are not evenly distributed throughout the tumor microenvironment. Some studies have reported greater homogeneity when examining multiple biopsies from the same tumor (20). Therefore, this finding evidences the importance of obtaining multiple sections and minimally culturing TILs, i.e., for 2–3 weeks, to accurately assess the immune landscape.

To evaluate if a higher infiltrate in certain areas was associated with elevated percentages of either CD4+ or CD8+ T cells, we analyzed the correlations between CD3+ percentages and CD8/CD4 ratios at both the outset and throughout the culture period. No clear association was found between the percentage of CD3+ T cells and the CD8/CD4 ratios in early explant cultures, indicating that both CD4+ and CD8+ T cells can coexist in areas with high lymphocytic infiltration. However, while the CD8/CD4 ratio remained relatively stable in early cultures, an inverse correlation emerged in cultures with CD8/CD4 > 1 over time. This suggests that CD4+ T cells, with a high proliferative nature, continue to expand in cultures even when CD8+ T cells are initially more abundant and therefore can dominate in long-term cultures.

We then examined the cytokines secreted during the early explant cultures (days 5, 10, 15, and 20) and correlated them with the percentages of CD3+, CD4+, and CD8+ T cells. The interpretation of cytokine data is complex due to the heterogeneity of TIL subpopulations and their capacity to secrete multiple cytokines. Additionally, different T cell subtypes can produce the same cytokines, complicating the attribution of specific responses to individual cell subsets. This underscores the importance of considering not only the percentages of CD4+ and CD8+ T cells but also the total CD3+ TILs to achieve a more comprehensive understanding of results obtained. Interpreting these results is more straightforward when analyzing cytokines that can be easily attributed to a specific cell subtype or effector mechanism. In contrast, cytokines that may be secreted by multiple subtypes need a more refined analysis, as the balance between these populations can influence their production. Therefore, the combined assessment of CD3, CD4, and CD8 patterns provides a more robust framework for understanding how interactions between these subpopulations affect the immune response in the tumor context.

For instance, in our cultures, we observed that a higher overall percentage of CD3+ T cells does not correlate with a more cytotoxic pattern; we only observed a significant negative correlation with certain cytotoxic molecules (sFasL and granzyme A). This indicates that either a greater presence of CD3+ T cells correlates with reduced cytotoxicity, or a lower presence of CD3+ T cells correlates with increased cytotoxicity. Importantly, this does not imply that a specific cell population is responsible; rather, in samples with high TIL content, the presence of non-cytotoxic cells such as some CD4+ T cells may mask the cytotoxic effect, as all measurements are based on correlations between cell percentages and cytokine secretion. Although we did not find a direct correlation between total CD3+ T cells and cytotoxic molecules, their pattern of association tended to follow that of CD8+ T cells, consistent with reports that higher T-cell infiltration is generally linked to better prognosis in many tumor types (21–24).

CD4+ T cell subtypes can have distinct roles in immunity, i.e., Th1 cells support cytotoxic responses through secretion of IL-2, TNF-α and IFN-γ, promoting activation of other effector T cells and antigen presentation (25, 26); Th2 generally promote tumor growth (27): and Th17 cells play dual roles, enhancing CTL recruitment via chemokine secretion but also promoting angiogenesis (28). In our cultures, we did not observe a strong association between CD4+ T cells and the secretion of hallmark cytokines defining Th patterns. This is probably due to the functional diversity within the CD4+ subset and the variety of cytokines they produce, complicating the establishment of a predominant profile.

Likewise, CTLs execute the lysis of target cells by releasing cytotoxic factors: perforins, which create pores in the membranes of (tumor) target cells, and granzymes and granulysin, which activate the caspase pathway, inducing apoptosis. CTLs also use other mechanisms mediated by the interaction of the FasL molecule (on the membrane of activated CTLs) with Fas (a death receptor on the membrane of the target cell). We observed that granulysin and granzyme A directly correlated with the presence of CD8+ T cells, while the percentage of CD4+ T cells exhibited an inverse correlation with most cytotoxic-related mediators, including sFasL, granulysin, granzyme A, granzyme B, and perforin. Thus, the most plausible conclusion is that, although the overall level of infiltration cannot be uniformly related to the cytotoxic response, there is a tendency for lower infiltration dominated by CD4+ T cells to correlate with reduced cytotoxicity. This pattern points to a link between the degree and composition of infiltration and the overall cytotoxic potential, while the underlying mechanisms remain to be elucidated.

On the other hand, we observed that an increase in the total CD3+ population correlates with a decrease in the IL-17A secretion, similar to the pattern observed with the increase in CD8+ T cells. However, this cytokine does not strongly correlate with the presence of CD4+ T cells nor with the CD8/CD4 ratios. Overall, this suggests that in regions with low TIL infiltration, a skewing of the CD8/CD4 balance towards CD4+ cells may be associated with higher IL-17A levels. This cytokine can be produced by various cell types (especially Th17, but also Treg or γδ T cells) and associated with both inflammation and regulation. The role of IL-17 in cancer has been extensively studied due to its plasticity (29–34). It has been described that IL-17 can promote tumor proliferation and induce angiogenesis by stimulating fibroblasts, which in turn increase the production of VEGF (vascular endothelial growth factor) (35). An increase in IL-17 has also been reported in BC (29, 36), and its presence has been associated with the recruitment of pro-tumor neutrophils, indicating a poorer prognosis (37). Recent studies in mouse models of colorectal cancer have shown that IL-17 inhibits the production of the chemokines CXCL9 and CXCL10 by tumor cells, reducing the recruitment of CD8+ T cells (38). This could provide an explanation for the inverse correlation observed between CD8+ T cells and the amount of IL-17, although further investigation is needed. Taking this into account, our data suggest a polarization between CD8+ T cells and Th17 cells, and the use of the CD8+/Th17 ratio could be useful, similar to the CD8+/Treg ratio.

Overall, our data indicate that explants with low overall infiltration and higher proportions of CD4+ T cells are associated with a clear inverse correlation with cytotoxic molecules, suggesting a limited effector function in these regions. These observations imply that, when selecting tumor sections for further study or potential therapeutic applications, regions with low TIL infiltration dominated by CD4+ T cells may be excluded. This highlights the importance of considering not only the degree of infiltration but also T cell subtype composition.

Finally, we performed TCR sequencing in our minimally cultured TILs as it is the molecule that confers antigen specificity to T cells; therefore, analyzing TILs TCRs can be used to identify tumor-specific sequences. Moreover, following T cell activation through recognition of p-MHC complexes by their TCRs, lymphocytes undergo clonal expansion. During clonal expansion, all lymphocytes express the same TCR, which allows monitoring of these clones through TCR sequencing.

We explored the characteristics of TCR CDR3 sequences and the overall TCR repertoire among minimally cultured TILs. No differences were observed in the analyzed properties based on tumor etiology (LA, LB, and TN) (data not shown). When examining the correlation patterns, we found that the overall percentages of CD3+ T cells aligned more closely with CD4+ T cell percentages. This observation might initially seem to diverge from our cytokine analysis. However, while cytokine levels reflect the activity of certain cells, TCR patterns illustrate the contributions of all TILs and subsets. Thus, TCR characteristics may be more influenced by the CD4+ subset, though this does not necessarily indicate that the effector response is predominantly driven by CD4+ T cells.

We observed significant correlations between specific CDR3 properties and the percentage of CD4+ T cells. Specifically, longer TRA CDR3 nt and shorter TRB N(D)N nt lengths positively correlated with the percentage of CD4+ T cells. Previous studies have also reported differences in CDR3 properties between T cell subsets. For instance, a previous study on peripheral T cells showed that CD8+ T cells tend to have longer CDR3 aa sequences (39), contrasting with our findings in minimally cultured TILs. Another study reported a higher frequency of negatively charged amino acids in CD8+ populations (40), a feature that we did not detect in our TIL samples.

Additionally, in the TCR repertoire analysis, higher percentages of CD4+ T cells were associated with lower diversity indices for both TRA and TRB sequences. This contrasts with previous reports, which indicate greater diversity in peripheral CD4+ T cells compared to CD8+ T cells (39, 40). Two different factors should be considered to explain the discrepancies with the literature. First, TILs represent a biased population, and results from studies using peripheral T cells are not directly comparable. Second, this study did not involve purified subpopulations: the correlations were drawn from flow cytometry percentages of CD4+ and CD8+ T cells and global properties derived from each culture. As such, some correlations may lack statistical significance due to the methodologies employed. However, in prior investigations conducted by our group using expanded and purified CD4+ and CD8+ TILs and comparing them with peripheral T cells from healthy donors, we also observed differences between subsets (13). These observations can help us to determine whether the differences are inherent to the subtypes or a product of an anti-tumor response.

The loss of diversity of CD4+ T cells could have two possible explanations: first, CD4+ TILs may exhibit a greater proliferative capacity in cultures, leading to reduced diversity. Second, these cells may arise from clonal expansions that occur *in situ* within the tumor. In our earlier study with expanded cells, we not only observed lower diversity in the CD4+ subset compared to CD8+, but also that CD4+ sequences tended to be more similar and shared more CDR3 motifs (13). This suggests that similar CD4+ CDR3 sequences are being selected during the anti-tumoral response, leading to uniform repertoire. This selection likely explains both the correlation of CDR3 nt lengths with the percentage of CD4+ T cells and the observed loss of diversity in this subset. In contrast, the greater diversity observed in CD8+ T cells may obscure any significant correlations with their CDR3 properties.

From a translational perspective, maintaining a representative and polyclonal TCR repertoire under minimal culture conditions is advantageous for subsequent therapeutic applications. Preserving repertoire diversity increases the likelihood of retaining tumor-reactive clones, which can later be identified or expanded in a controlled manner. In early cultures derived from high-TIL sections, CD4+ T cell diversity remained high even when their relative abundance was lower, likely supported by the concomitant presence of abundant CD8+ T cells. In contrast, low-TIL explants consistently exhibited reduced CD4+ diversity, and CD8+ T cells from these sections also displayed constrained repertoire diversity. Our findings underscore the importance of selecting explants from highly infiltrated sections to preserve a diverse and representative TCR repertoire and that applying minimal culture conditions maintains both CD4+ and CD8+ T cell repertoires. Altogether, this would maximize the likelihood of capturing tumor-specific TCRs, thereby enhancing the potential efficacy of subsequent TIL-based therapies. Although our study did not include functional validation or clinical correlation, these repertoire data provide a crucial foundation for selecting and optimizing explant-derived TILs for ACT approaches.

Unlike conventional rapid-expansion protocols using strong stimulation and high-dose IL-2, which can drive T cells toward terminal differentiation or exhaustion, our study underscores the importance of minimally culturing TILs, based solely on tumor tissue and external low-dose IL-2, to preserve their original characteristics and functionality, which was further validated by the correlation with the IHC analysis. Regions with low TIL infiltration showed a higher presence of CD4+ T cells, which was inversely associated with the secretion of cytotoxic mediators, and showed reduced TCR diversity. In contrast, sections displaying a more balanced representation of CD4+ and CD8+ TILs were generally indicative of highly infiltrated regions and exhibited an overall higher repertoire diversity. These observations emphasize that the co-existence of both subsets, rather than the dominance of one, is important for sustaining an effective antitumor response and should be considered when optimizing immunotherapeutic strategies.

Moreover, our minimally expanded TIL approach mirrors the “young TIL” strategy employed in ACT trials, where unselected, bulk TILs derived from multiple tumor fragments are minimally cultured (41, 42). This strategy allows faster delivery of TIL therapy to eligible patients and has demonstrate to mediate regression of certain tumors (43, 44). Similarly, our minimally culture method simplifies TIL selection allowing a broader accessibility while preserving functional diversity. Other methods rely on the selection of TILs expressing certain surface markers such as PD-1, CD39 or CD103, or CD137 (45–51), which can reduce overall cell yield and bias selection toward exhausted or terminally differentiated clones (52–55), potentially excluding less-differentiated, tumor-reactive clonotypes. By avoiding such bias, our method preserves polyclonality and allows subsequent functional and TCR-based characterization, which may be critical for effective antitumor responses.

Overall, these findings provide a comprehensive view of the interplay between TIL subset composition, infiltration patterns, and functional potential, highlighting strategies to optimize TIL-based therapies. Maintaining a minimally exhausted, polyclonal repertoire may enhance clinical efficacy, and integration with current immunotherapeutic approaches could improve accessibility and outcomes in breast cancer treatment.

## Data Availability

The authors selected the following statement: The datasets presented in this study can be found in online repositories. The names of the repository/repositories and accession number(s) can be found below: https://www.ncbi.nlm.nih.gov/, PRJNA925311, https://www.ncbi.nlm.nih.gov/, PRJNA759174.
