# Supplementary material for: Minimally expanded breast cancer tumor-infiltrating-lymphocytes provide guidance for therapeutic selection
Source: Front Immunol. 2025 Nov 26;16:1699262. doi: 10.3389/fimmu.2025.1699262 (PMC12689370; doi:10.3389/fimmu.2025.1699262)

Supplementary Figures

**Supplementary Figure 1. Percentages of CD3+ lymphocytes and CD8/CD4 ratios in the biopsies and sections analyzed during the culture weeks.** CD3+ percentages (in green) and CD8/CD4 ratios (from red to blue, with red representing CD8/CD4<1 and blue representing CD8/CD4>1) obtained by flow cytometry. The week of staining and the different sections of each studied biopsy are shown in order: (A) 562, (B) Q1, (C) Q2, (D) Q7, (E) Q8, (F) Q10, (G) Q12, (H) Q14, (I) Q15, (J) Q16, and (K) Q17. Boxes with an X indicate that staining could not be performed for that section on that date.


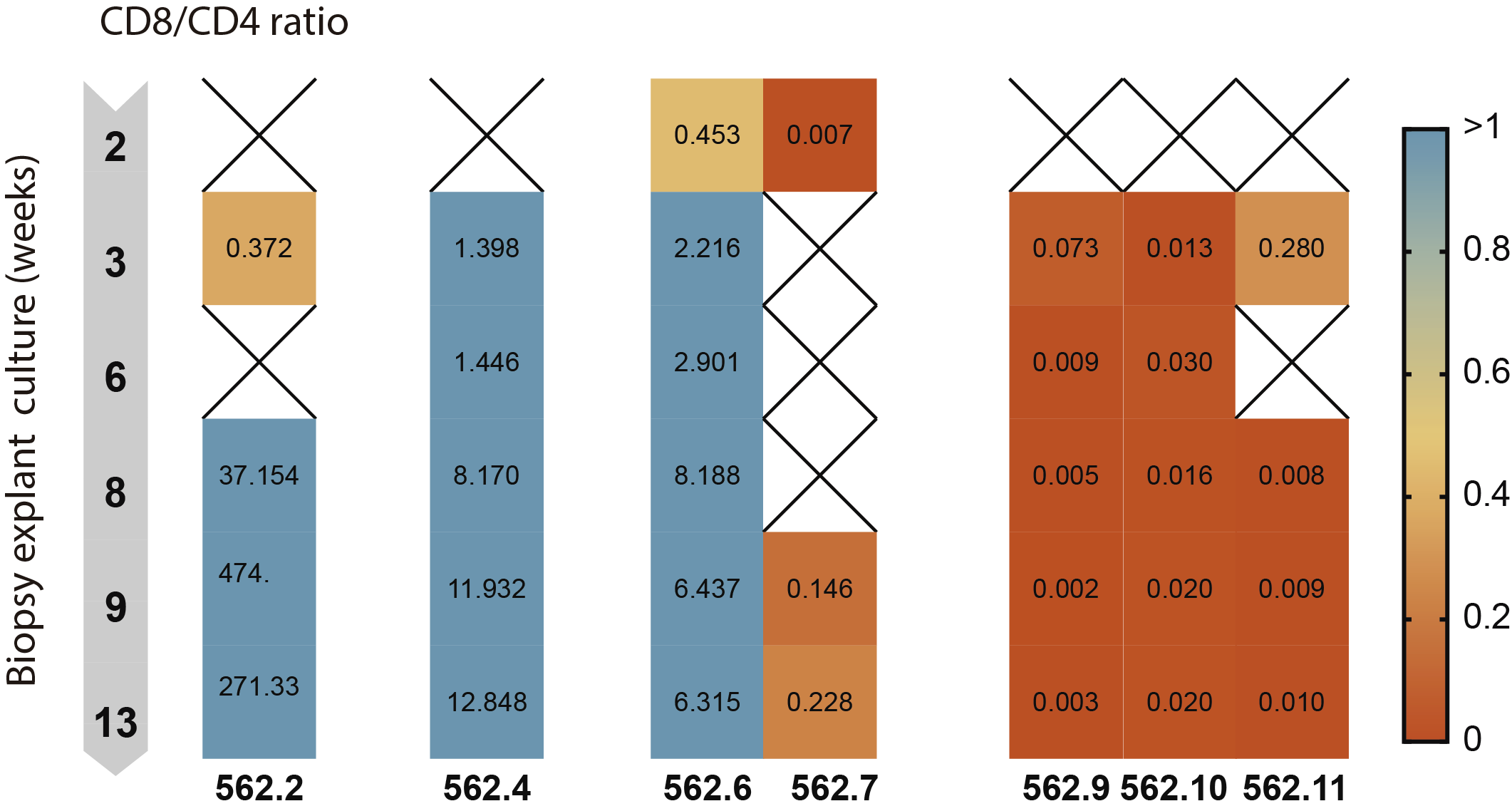

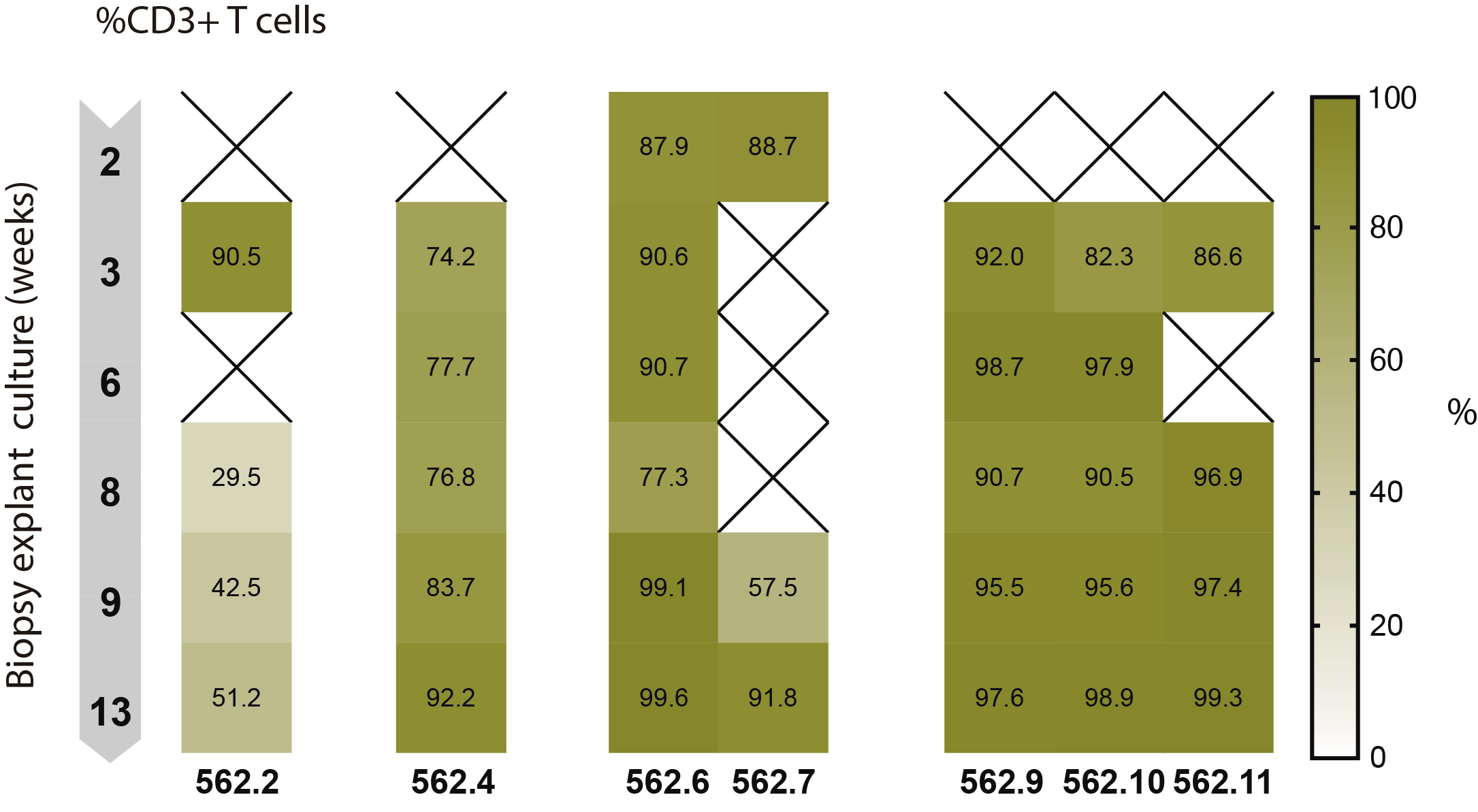

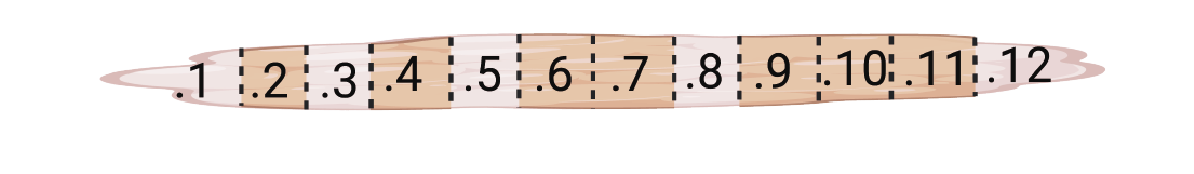


**A BC-PS-562**


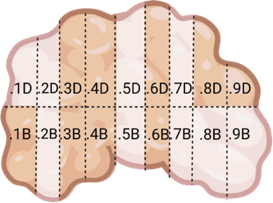

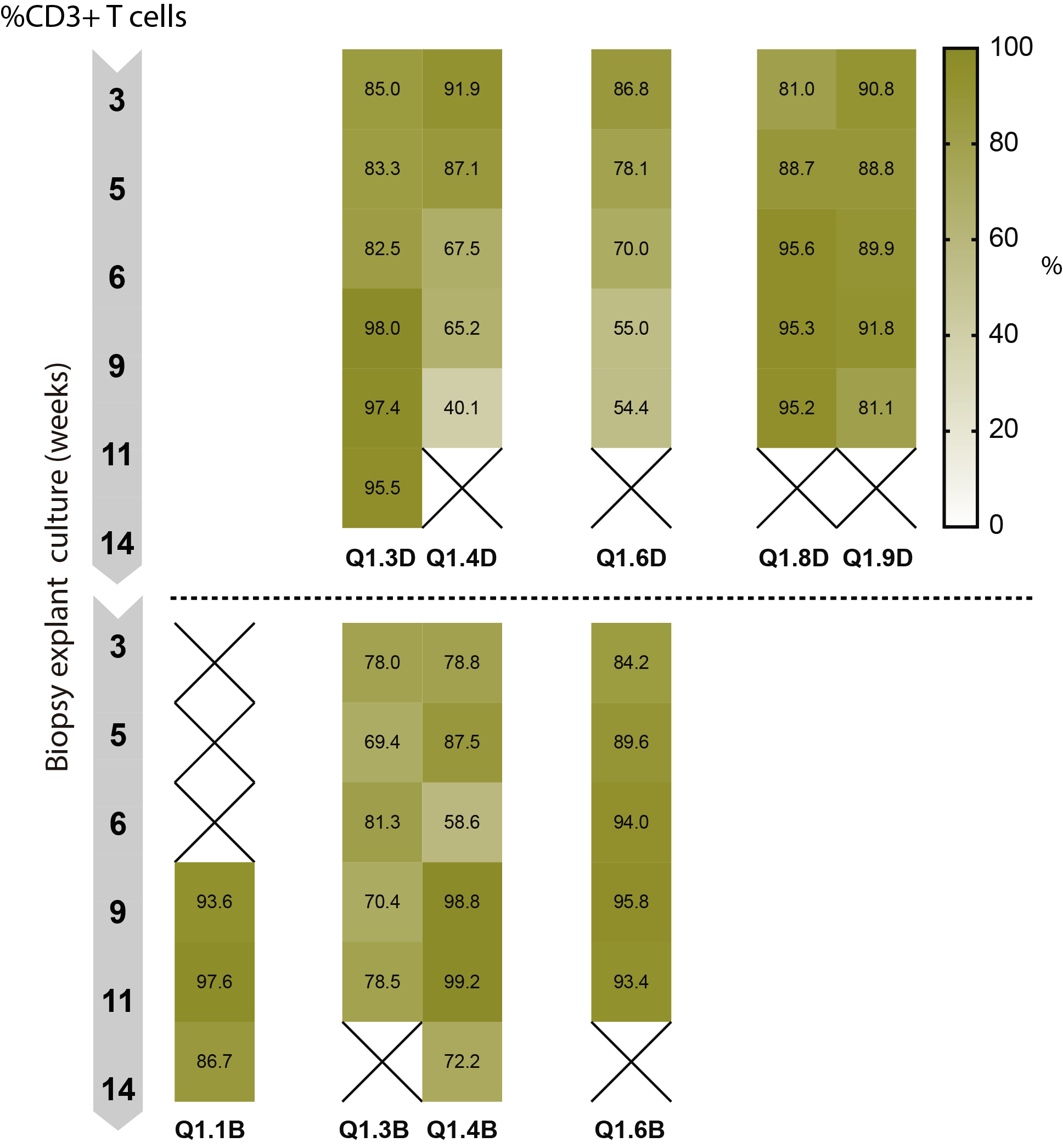

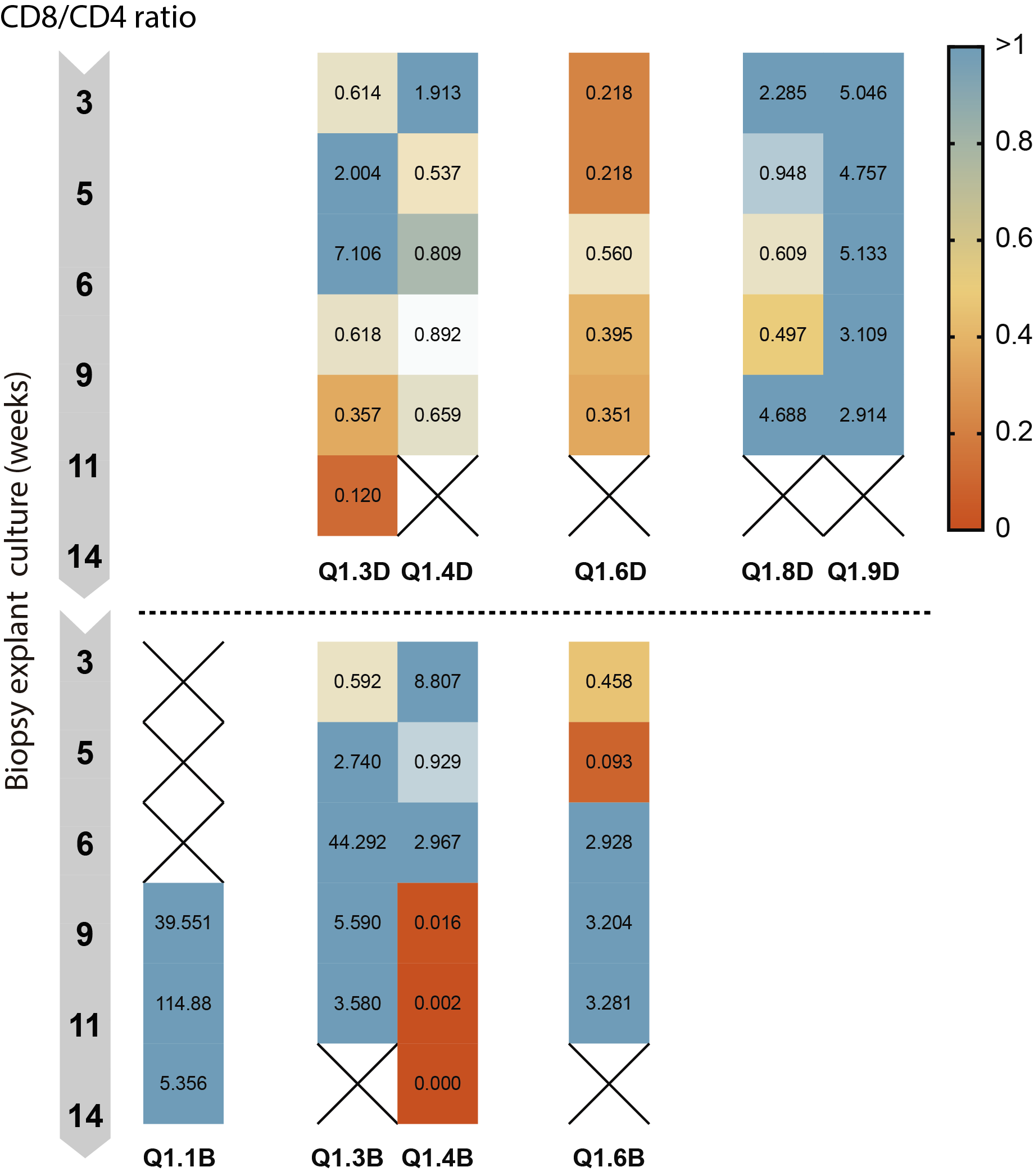


**B BTLQ1**


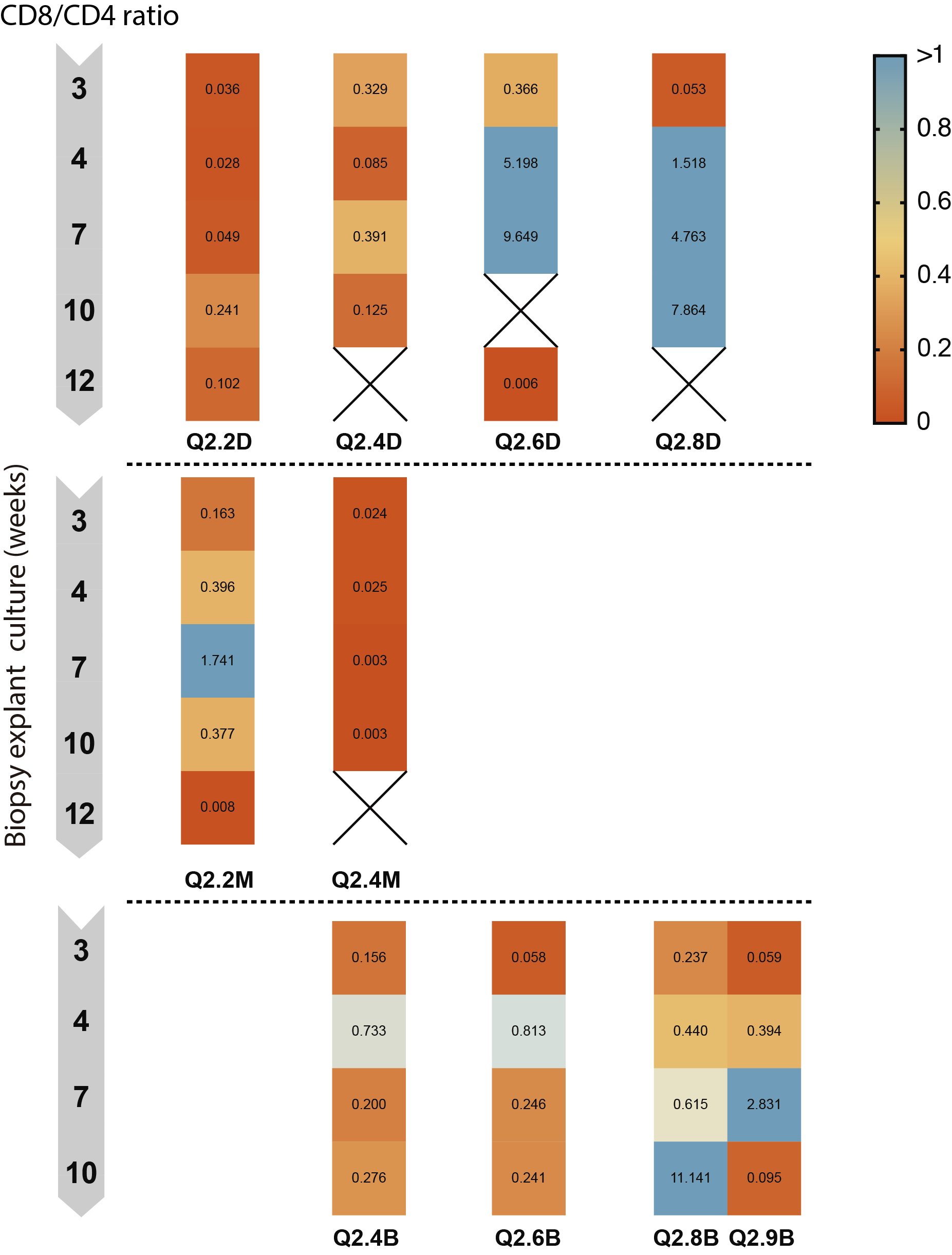

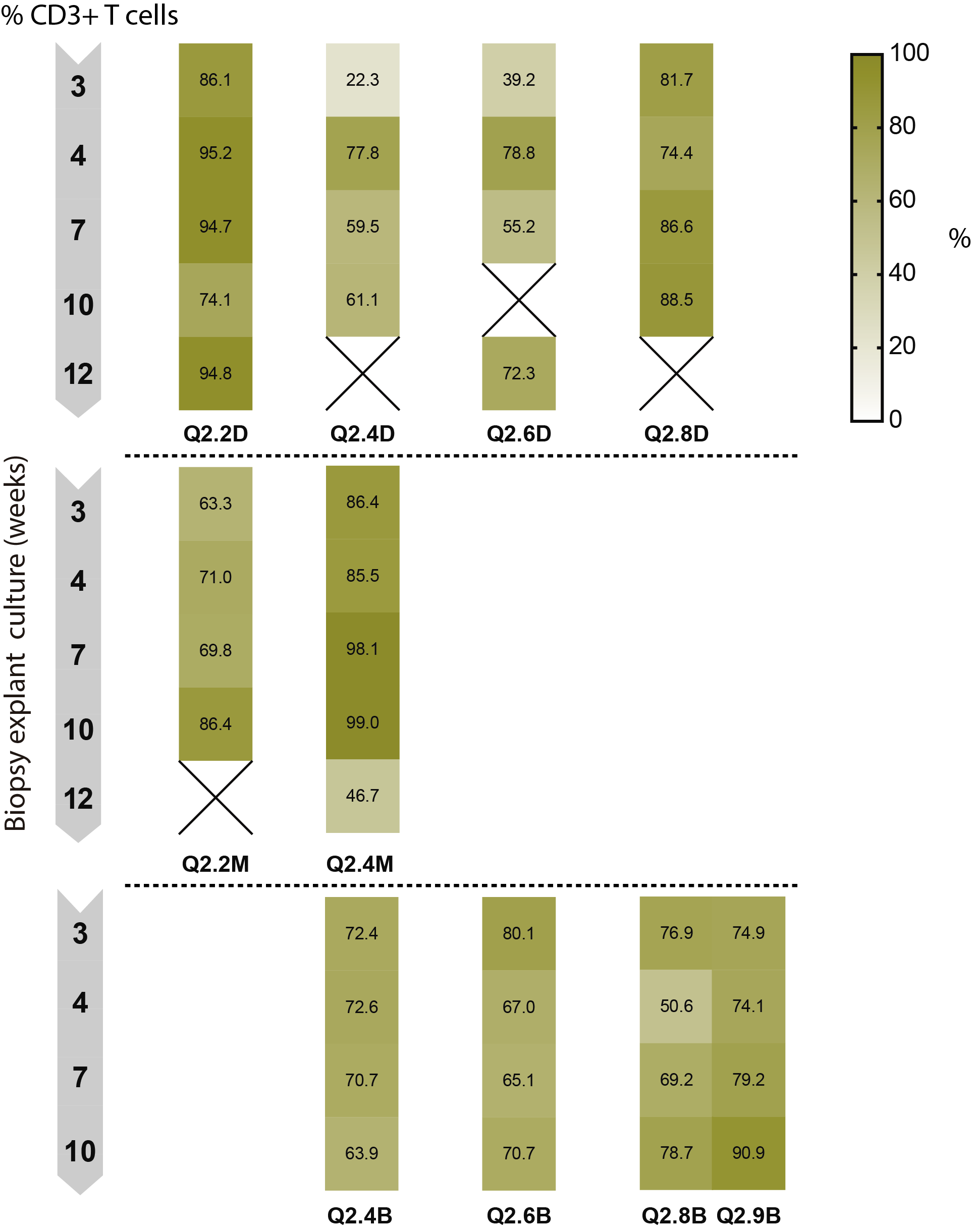

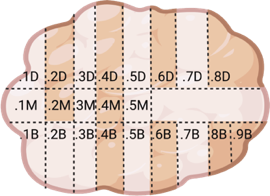

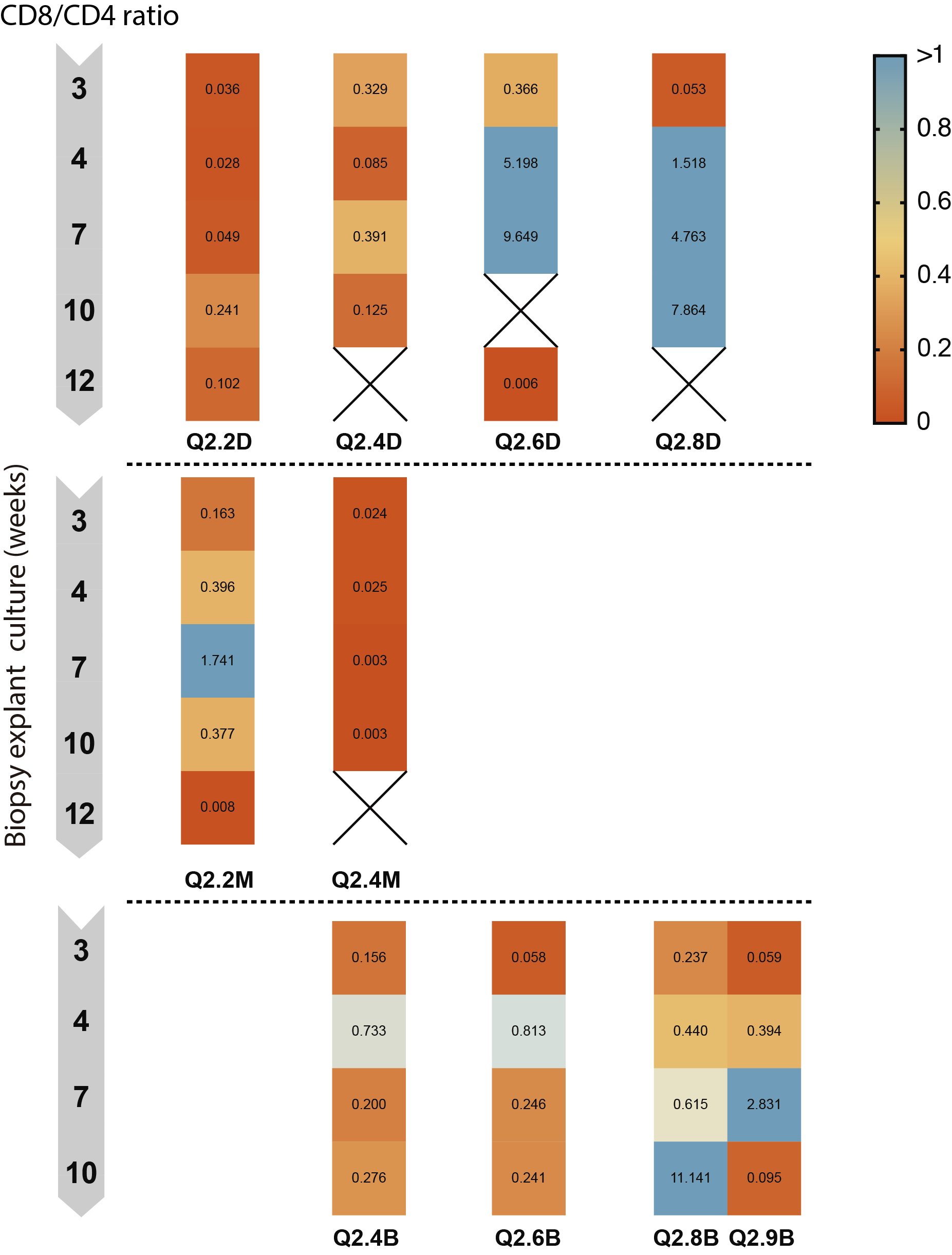

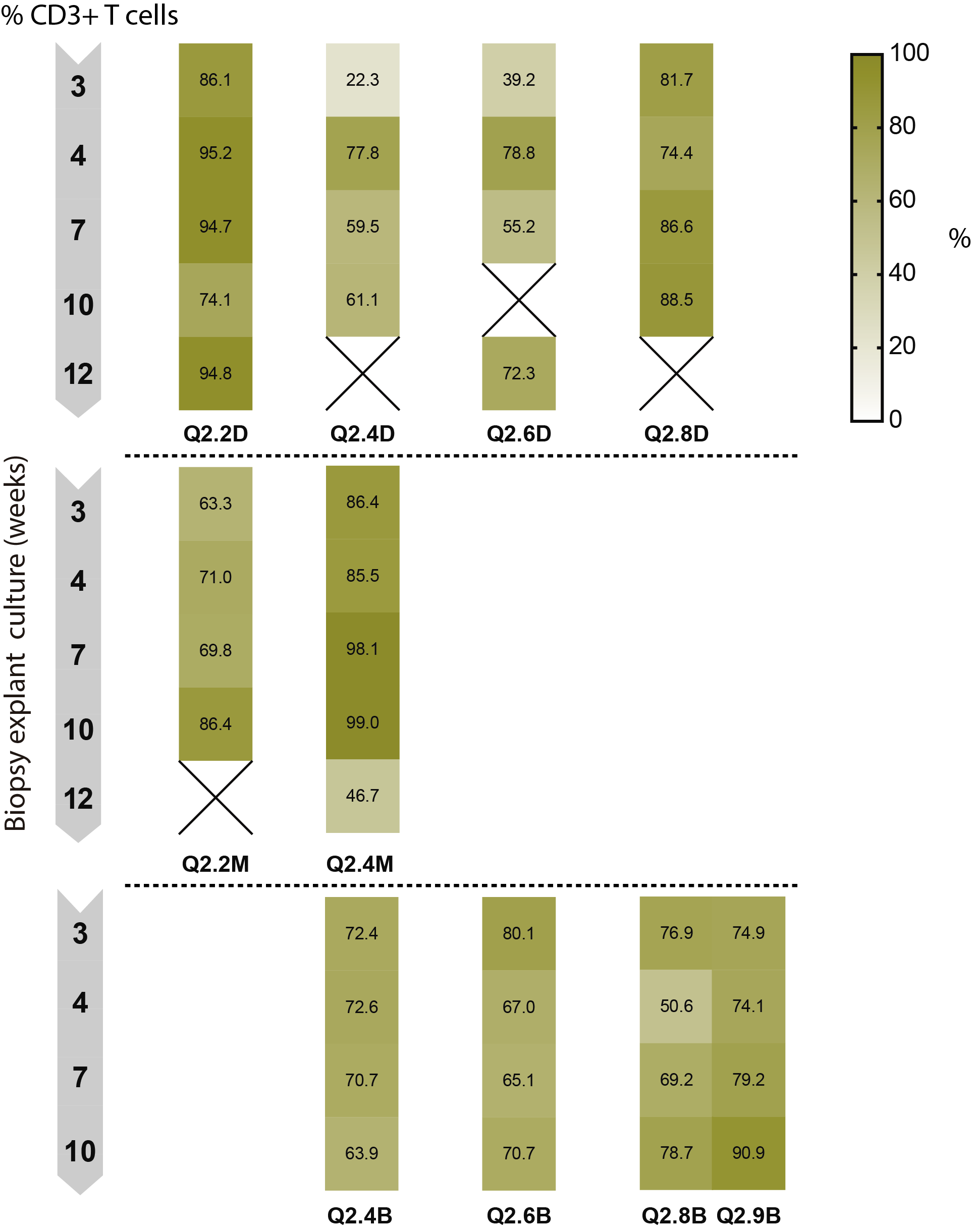


**C BTLQ2**


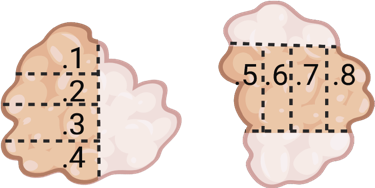

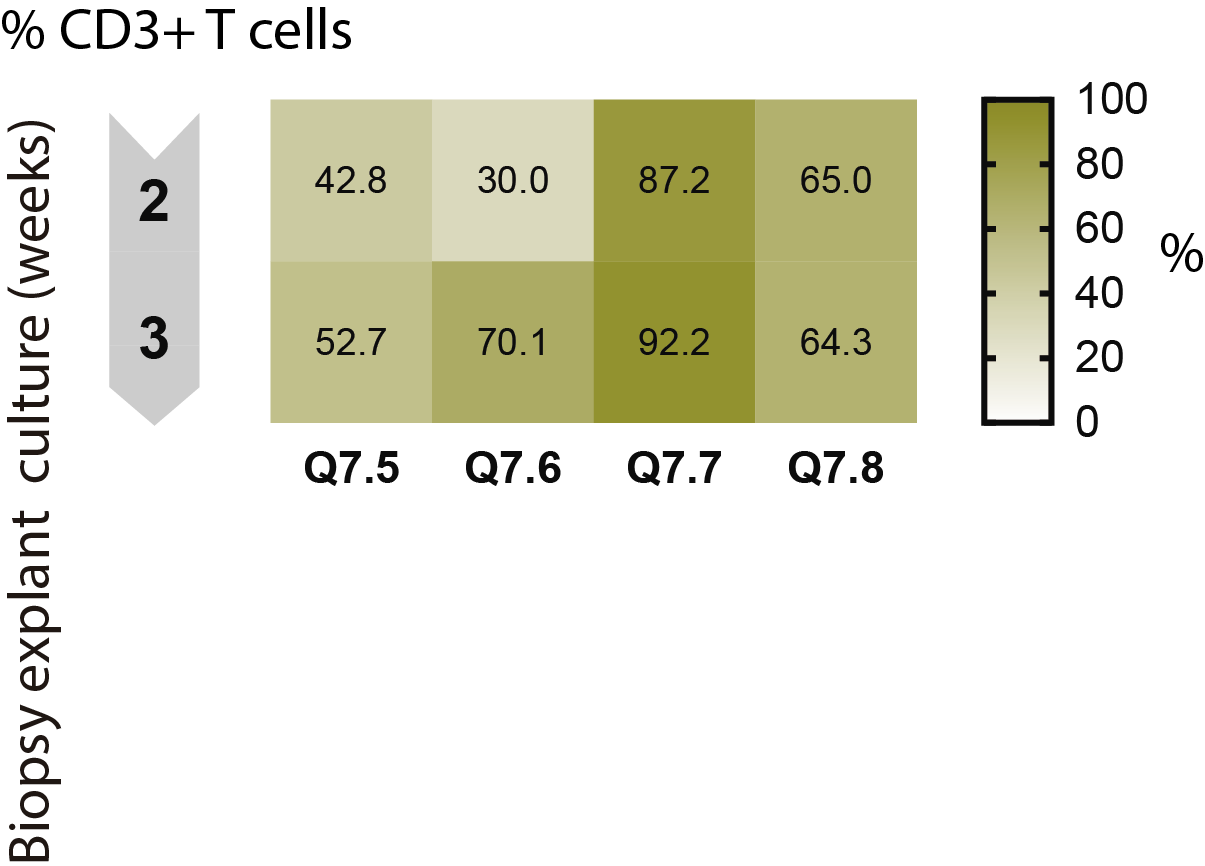

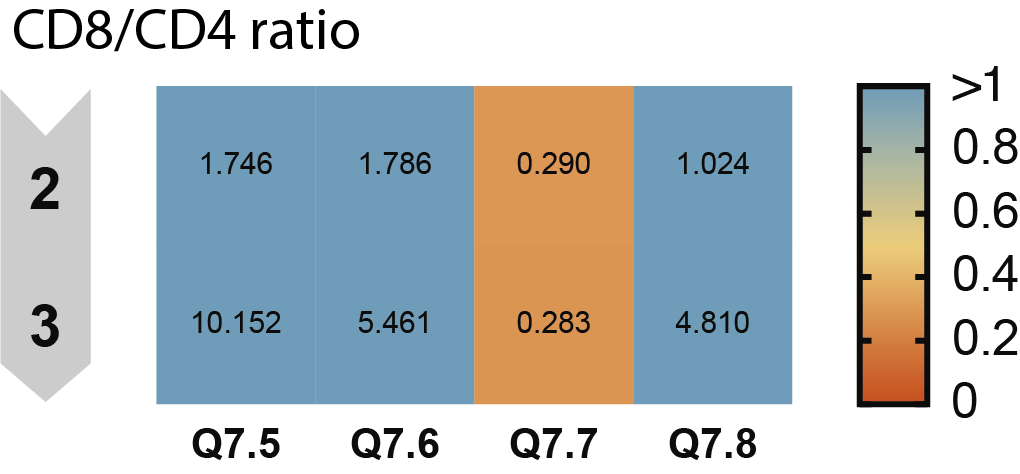

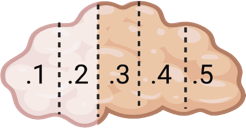

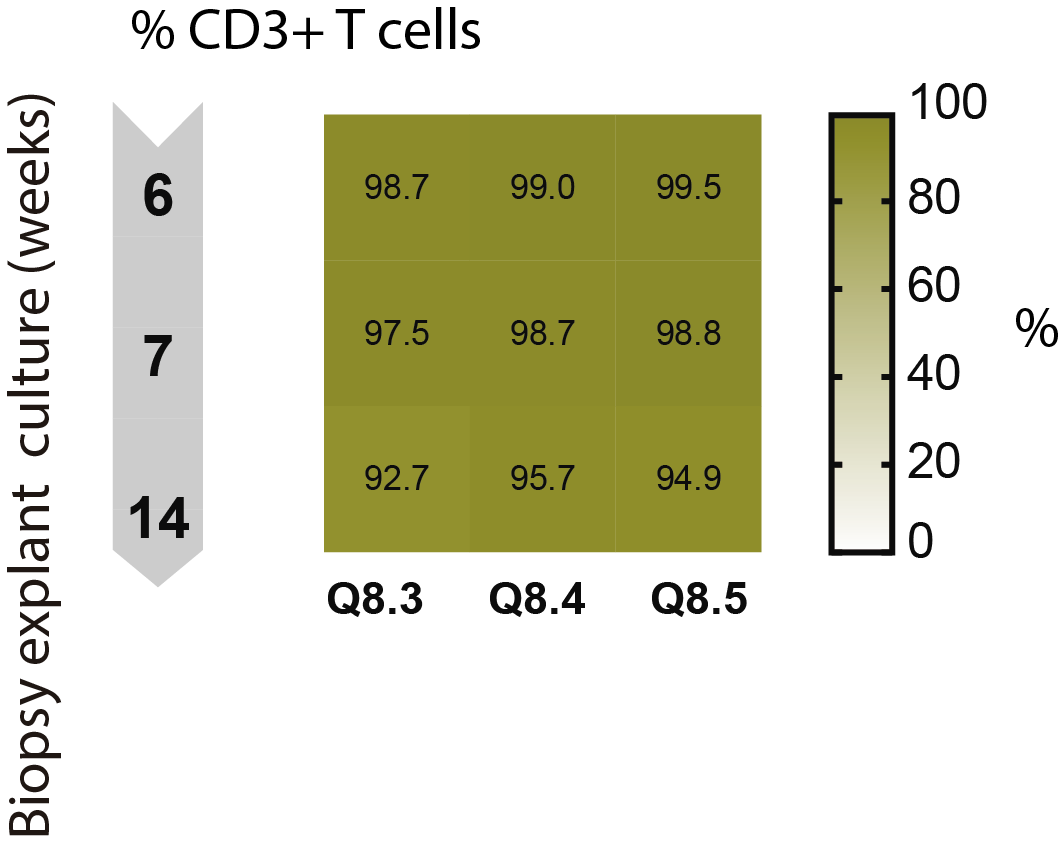

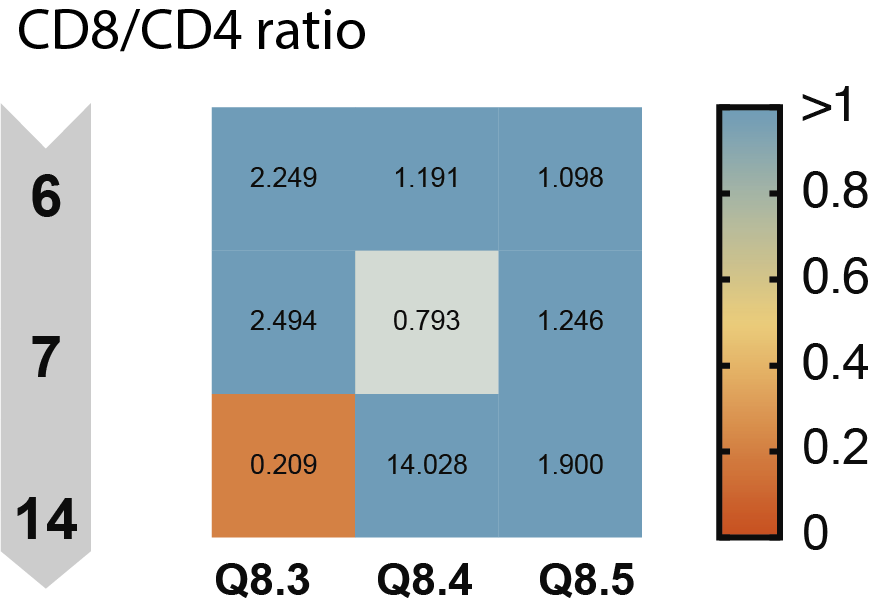

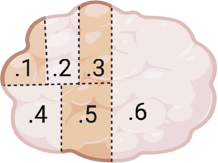

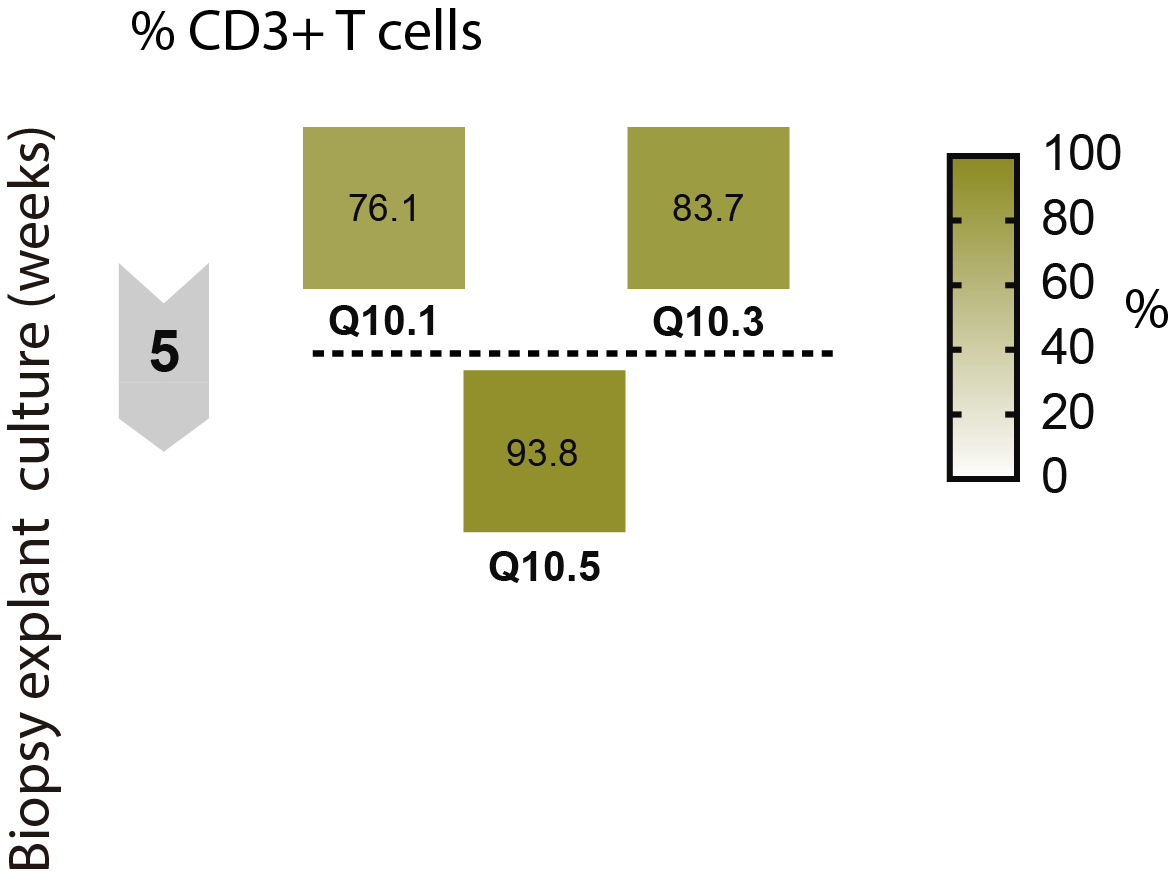

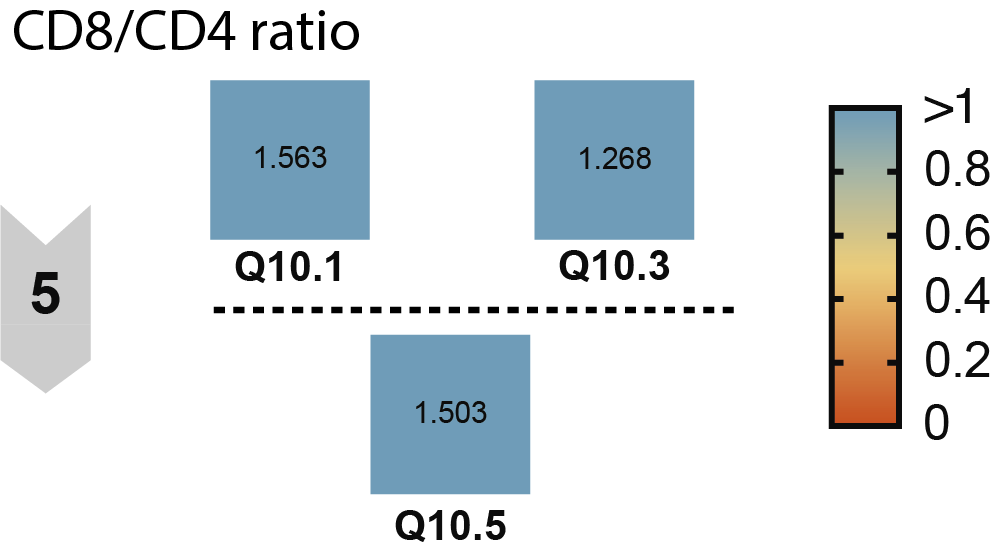


**D BTLQ7 E BTLQ8 F BTLQ10**


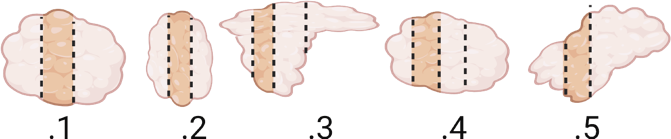

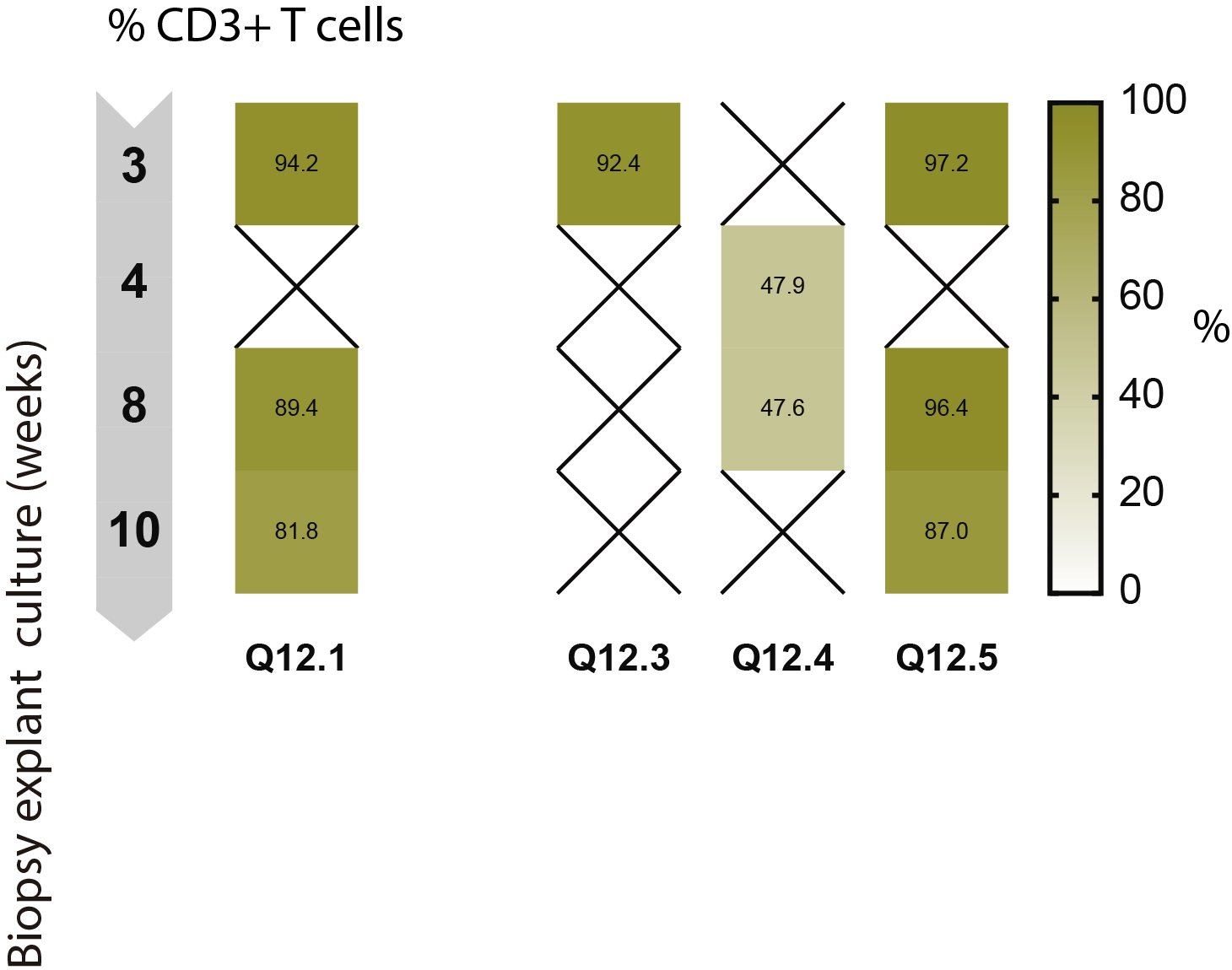

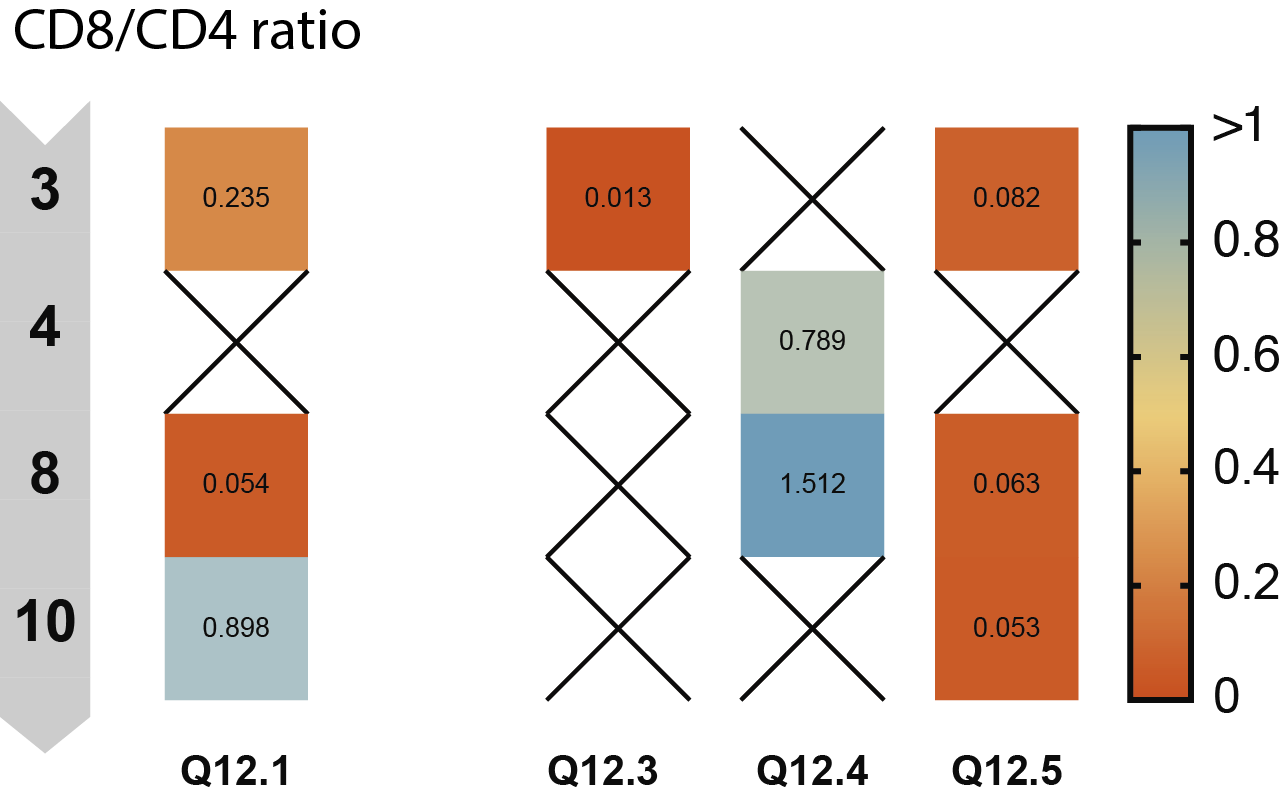

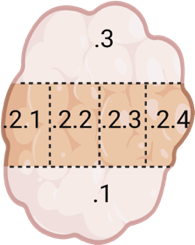

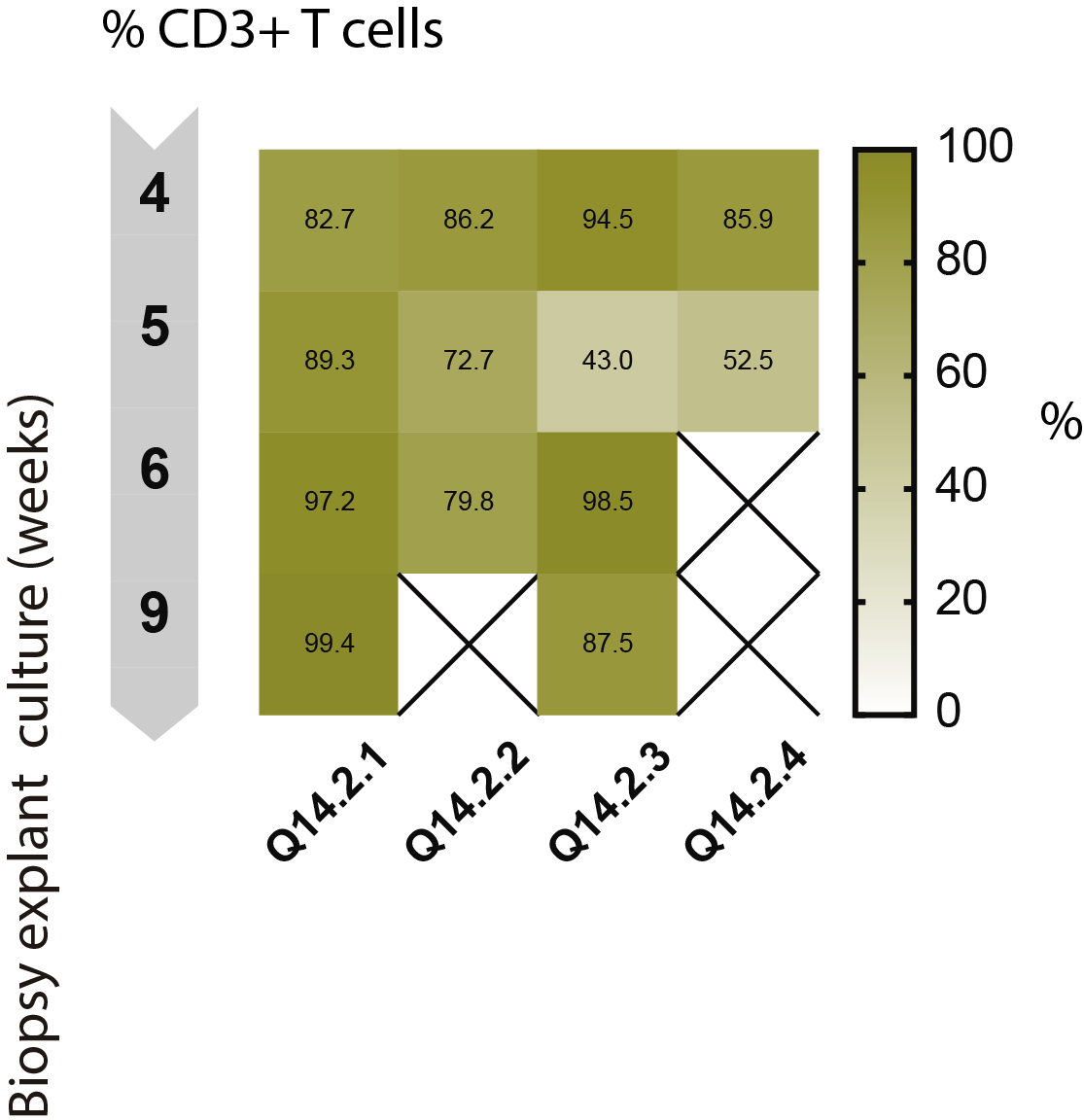

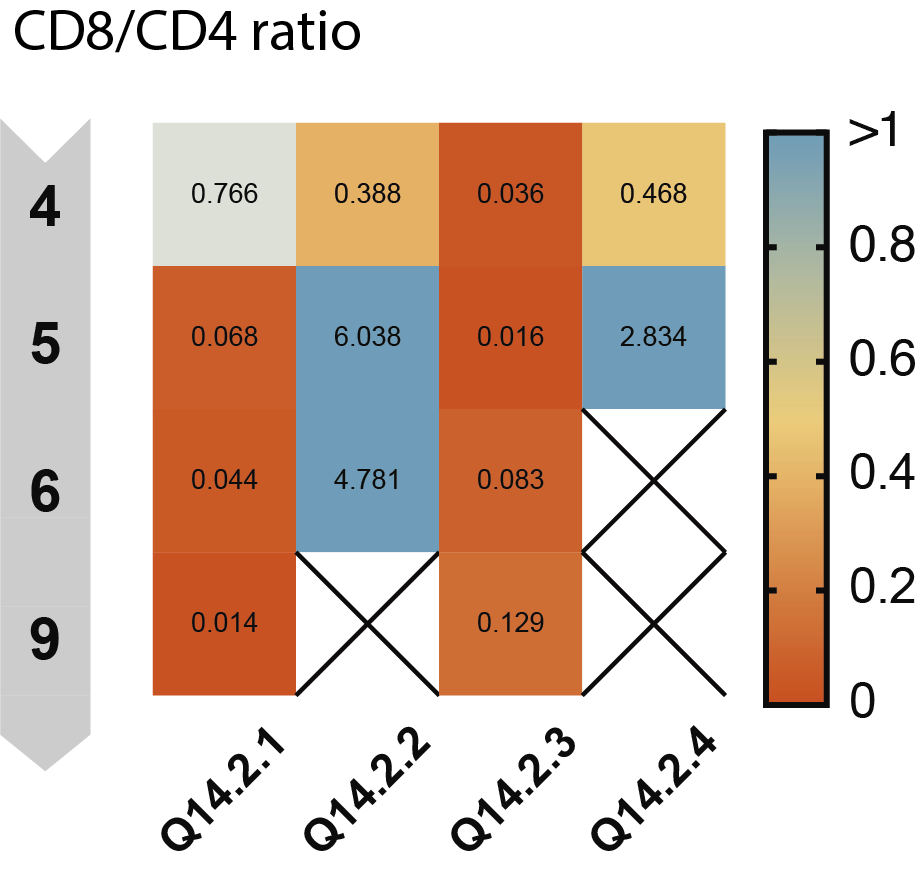


**G BTLQ12 H BTLQ14**


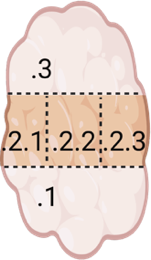

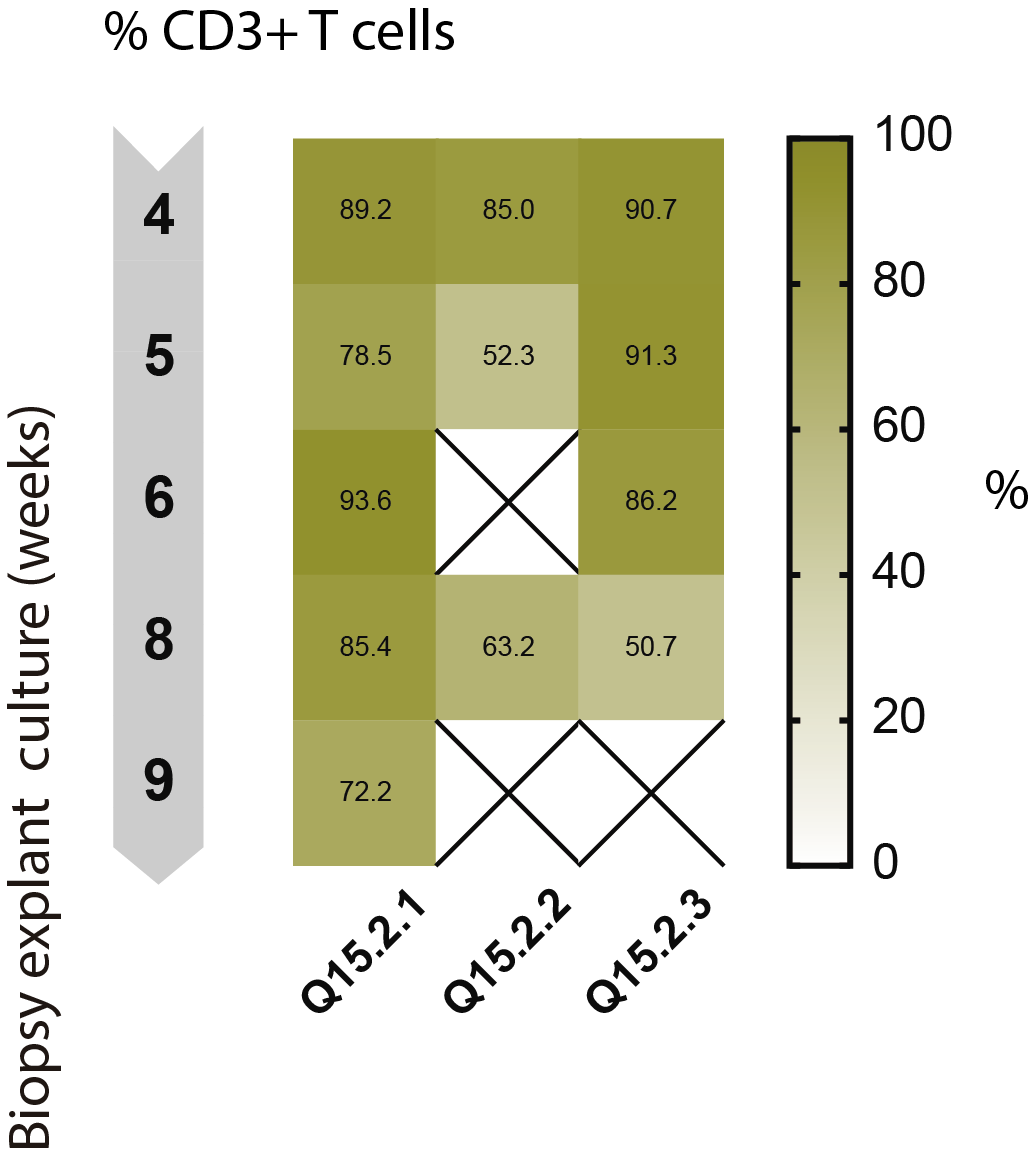

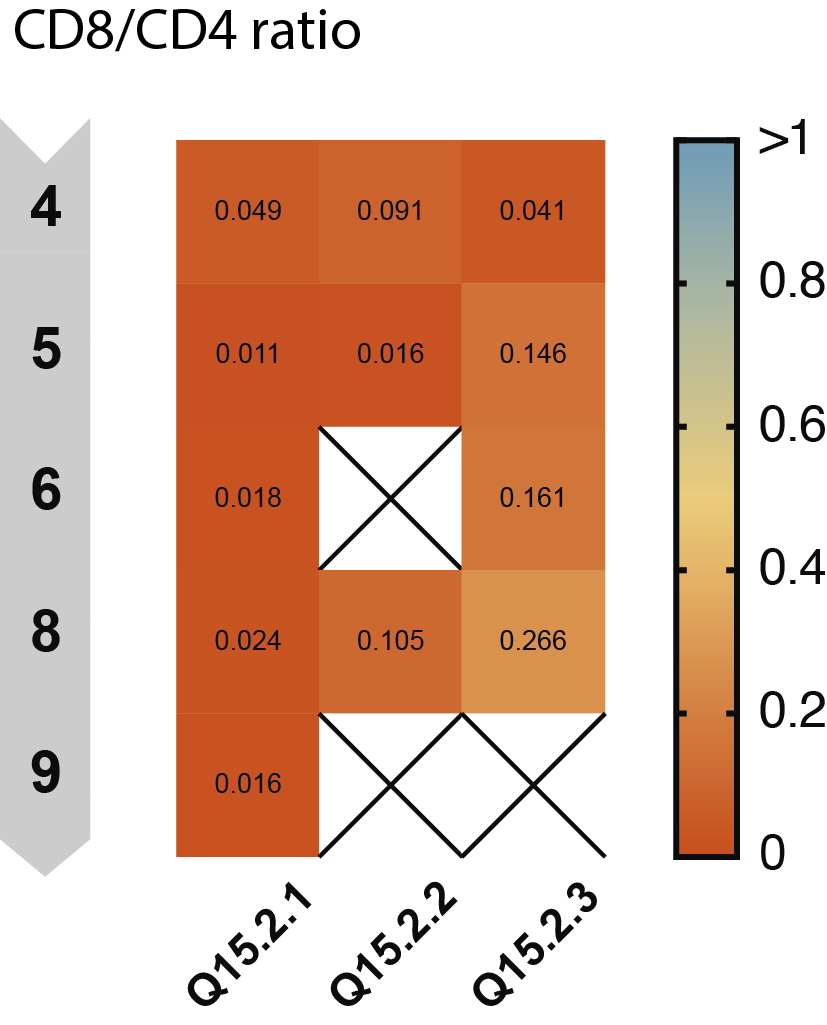

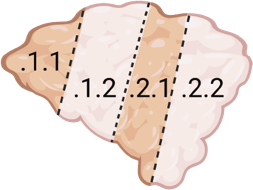

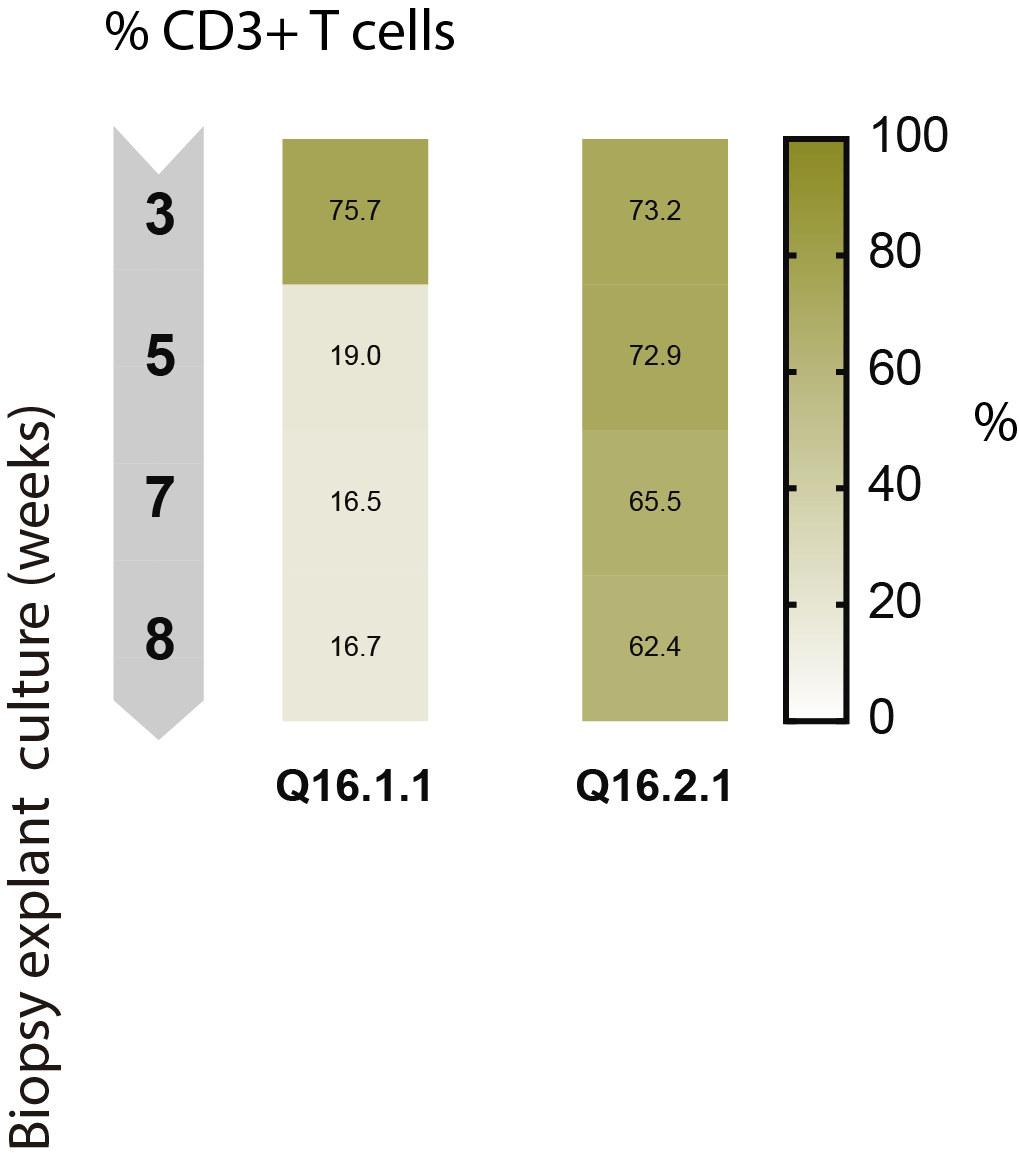

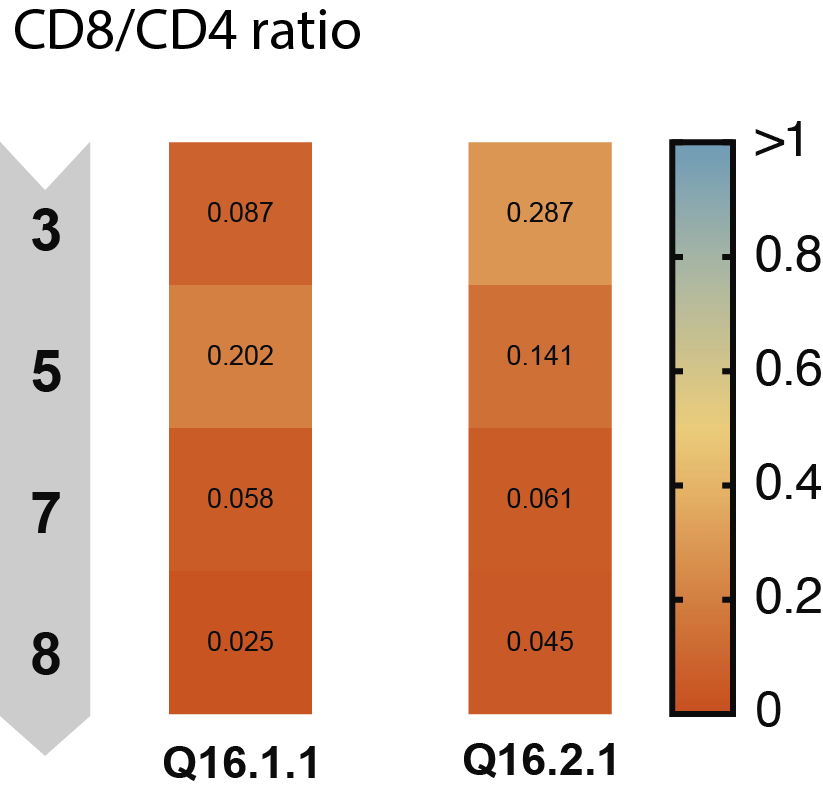

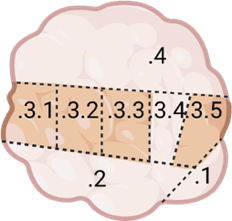

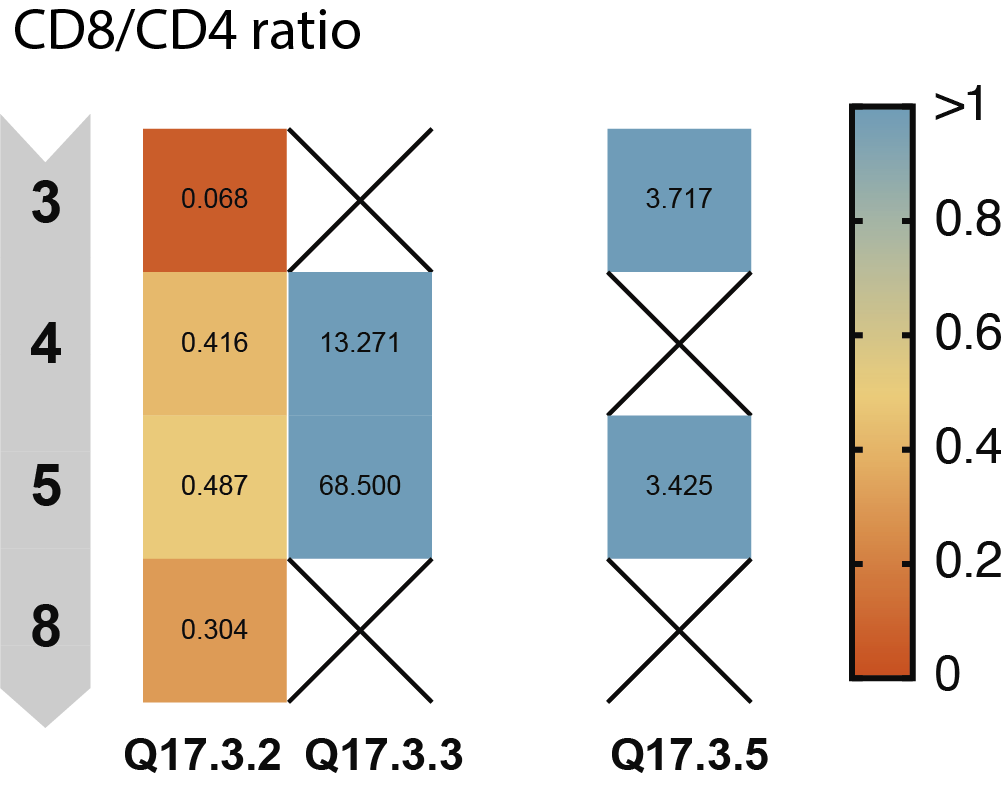

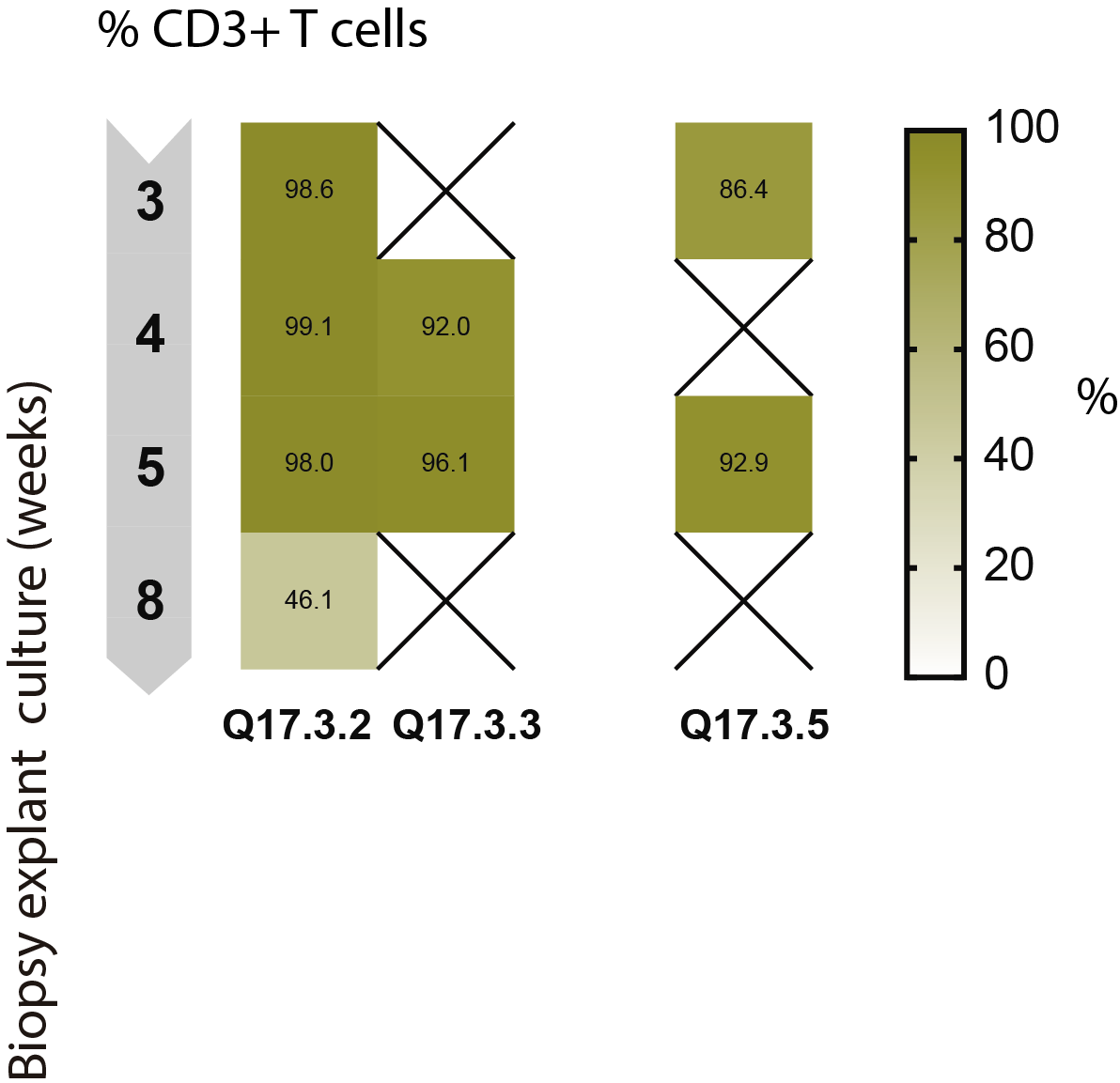


**I BTLQ15 J BTLQ16 K BTLQ17**

Supplementary Figure 2. Correlation between secreted molecules in the supernatant and the percentage of CD3+ TILs in the initial cultures. The correlation between the quantity of secreted molecules (in pg/ml) and the percentages of CD3+ TILs in the culture on the same date as the supernatant collection was studied using linear regressions. An inverse correlation was observed between the percentage of CD3+ TILs and the amount of IL-17A (p = 0.017), sFasL (p = 0.039) and granzyme B (p = 0.02).


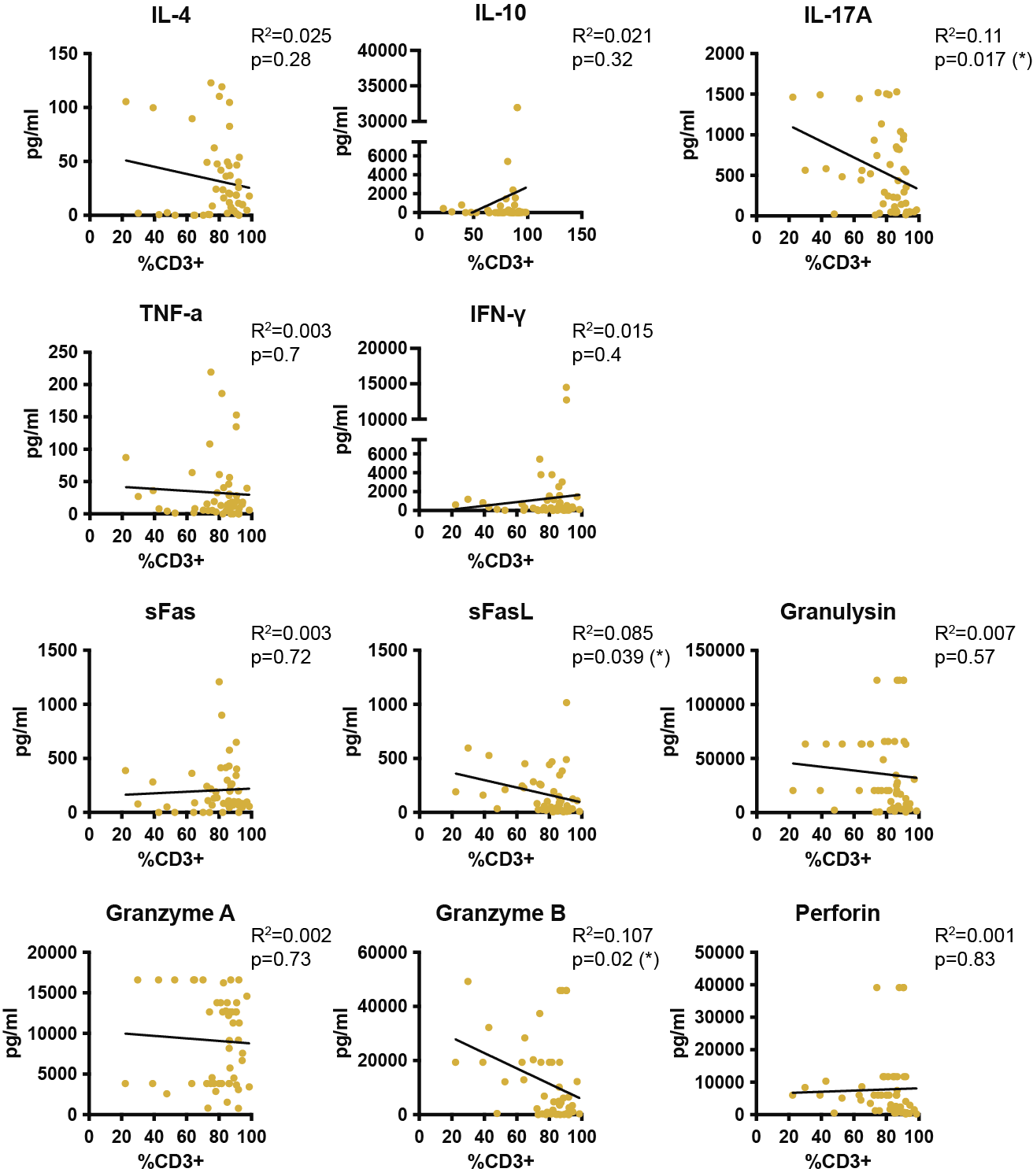


Supplementary Figure 3. Correlation between secreted molecules in the supernatant and the percentage of CD4+ TILs in the initial cultures. The correlation between the quantity of secreted molecules (in pg/ml) and the percentages of CD4+ TILs in the culture on the same date as the supernatant collection was studied using linear regressions. An inverse correlation was observed between the percentage of CD4+ TILs and the amount of sFasL (p = 0.0006), granulysin (p < 0.0001), granzyme A (p = 0.001), granzyme B (p = 0.0098), and perforin (p = 0.0026).


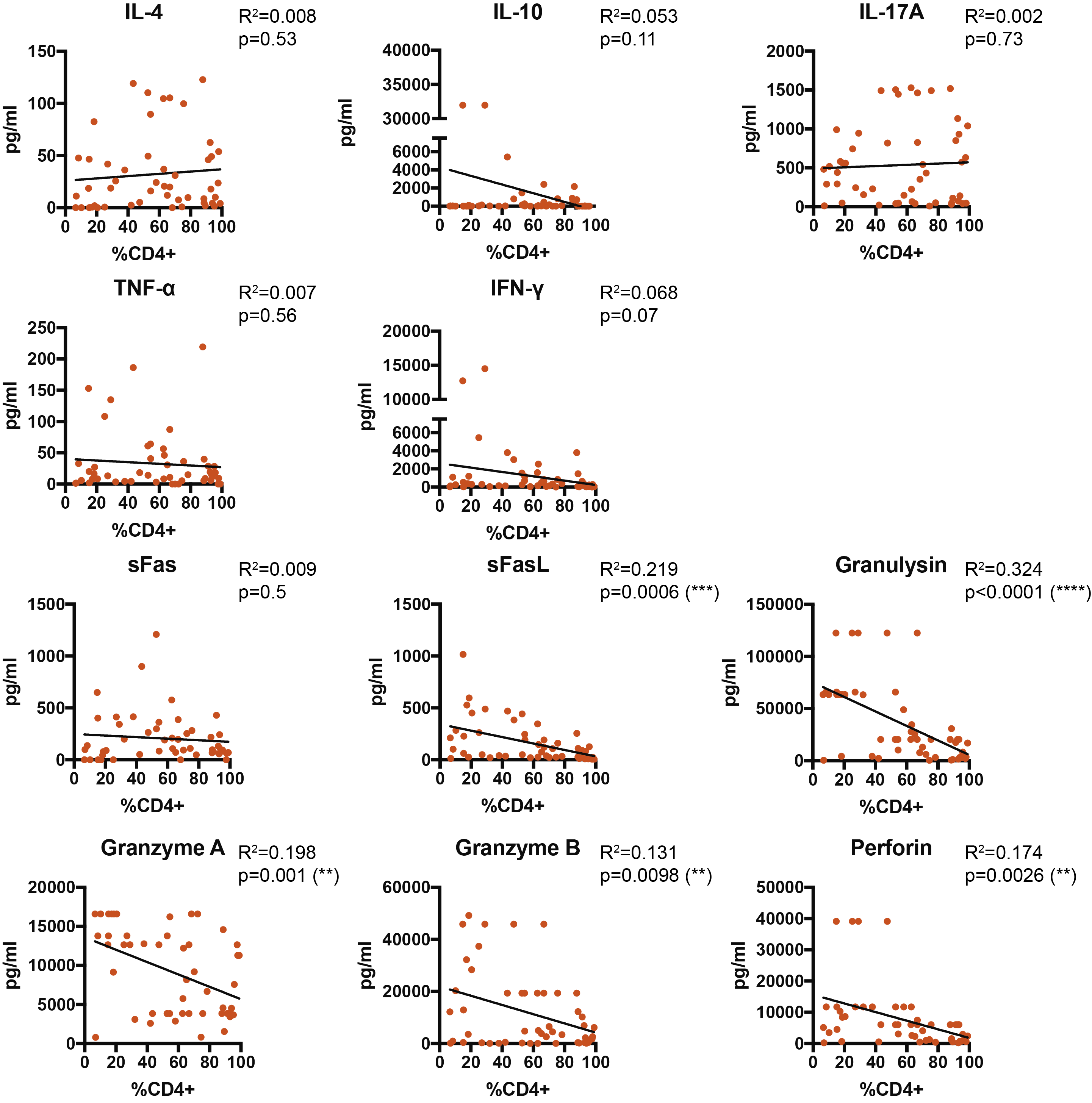


Supplementary Figure 4. Correlation between secreted molecules in the supernatant and the percentage of CD8+ TILs in the initial cultures. The correlation between the quantity of secreted molecules (in pg/ml) and the percentages of CD8+ TILs in the culture on the same date as the supernatant collection was studied using linear regressions. An inverse correlation was observed between the percentage of CD8+ TILs and the amount of IL-17 (p = 0.018), as well as a direct correlation with the production of granulysin (p = 0.025) and granzyme A (p = 0.029).

**
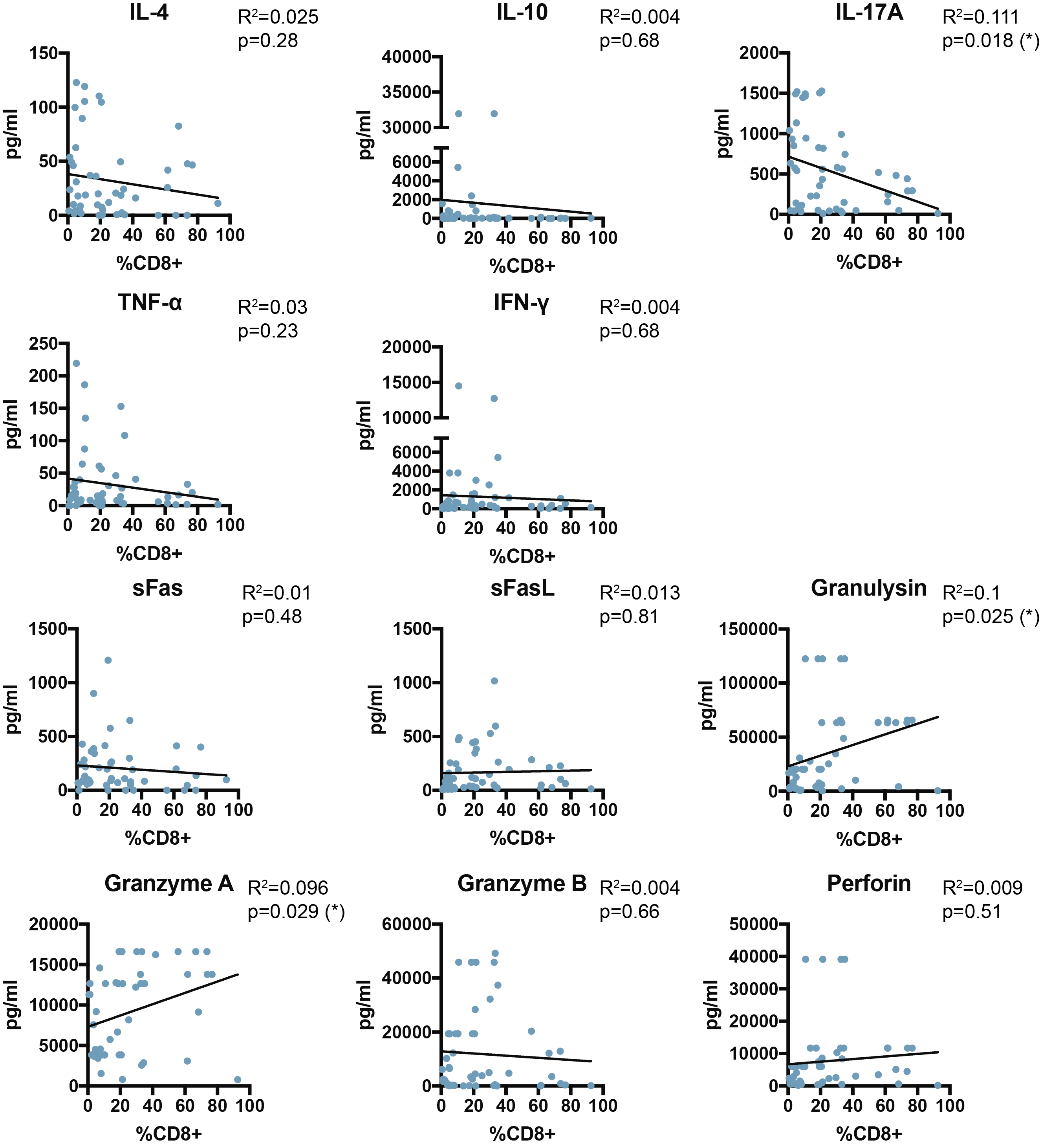
**

Supplementary Figure 5. Correlation between secreted molecules in the supernatant and the CD8/CD4 ratios in the initial cultures. Correlation was studied, using linear regressions, between the quantity of secreted molecules (pg/ml) and the CD8/CD4 ratios in the culture on the same date as the supernatant collection. No correlation was observed.


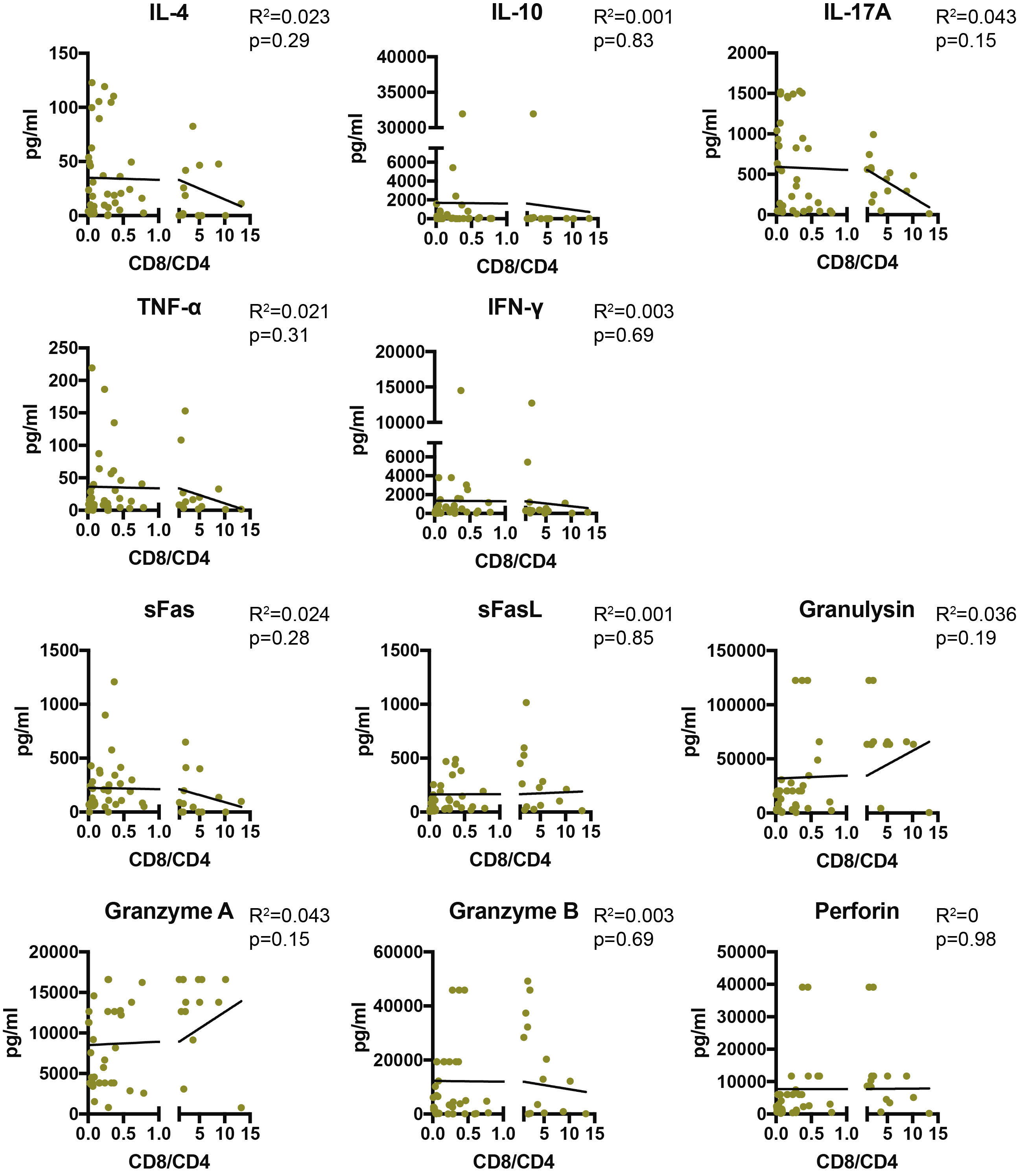


**Supplementary Figure 6. TCR richness in the different samples of each biopsy.** (A), (B) Rarefaction curve of each biopsy for TRA and TRB sequences, showing the number of distinct clones (richness) relative to the sample size (in number of reads) using Immunarch^16^. Dashed lines indicate extrapolation to the largest sample. (C), (D) Number of TRA CDR3nt and TRB CDR3nt sequences in the different sections (represented by dots) of the analyzed biopsies (bars represent the mean of each biopsy).

**
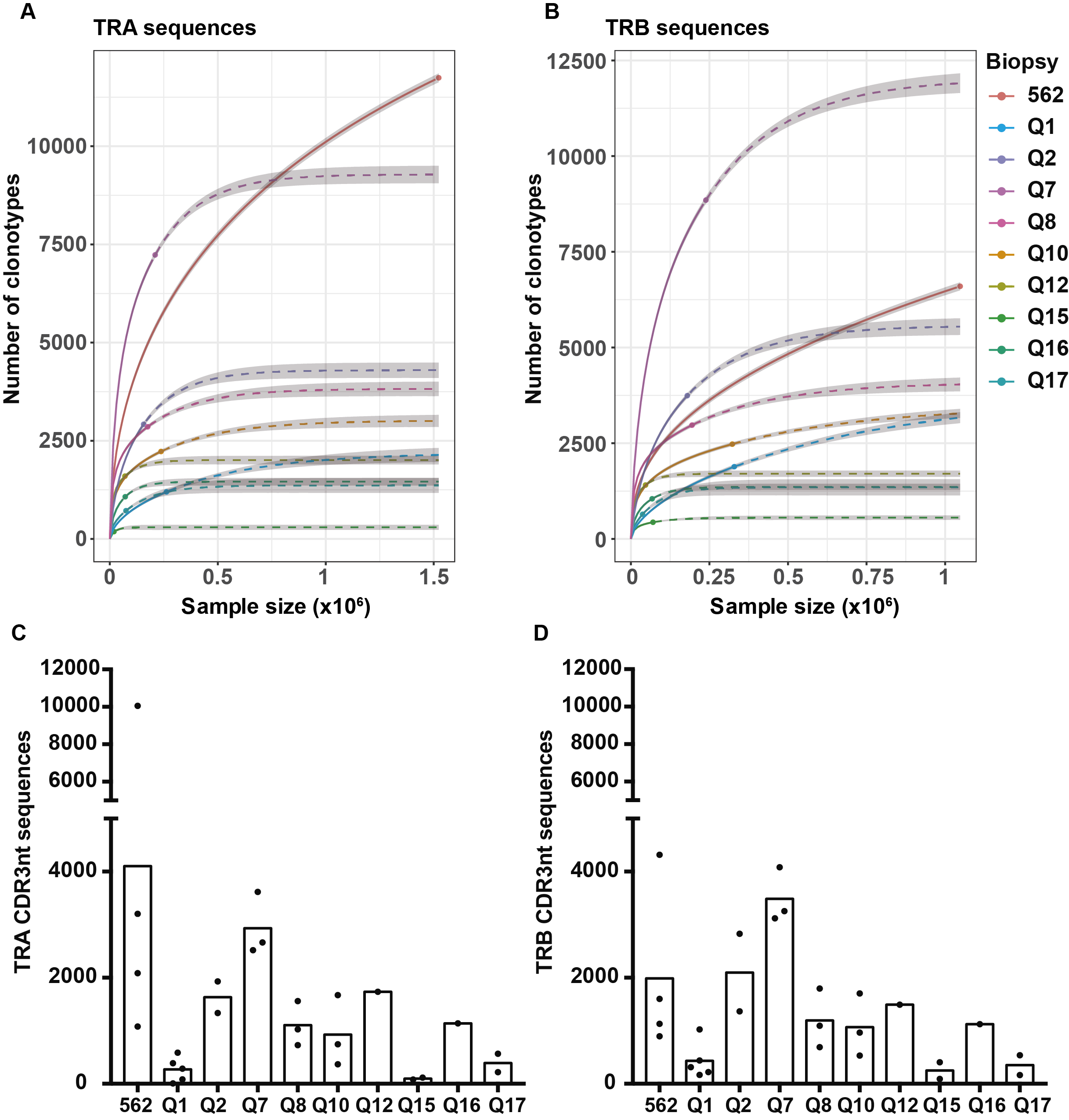
**


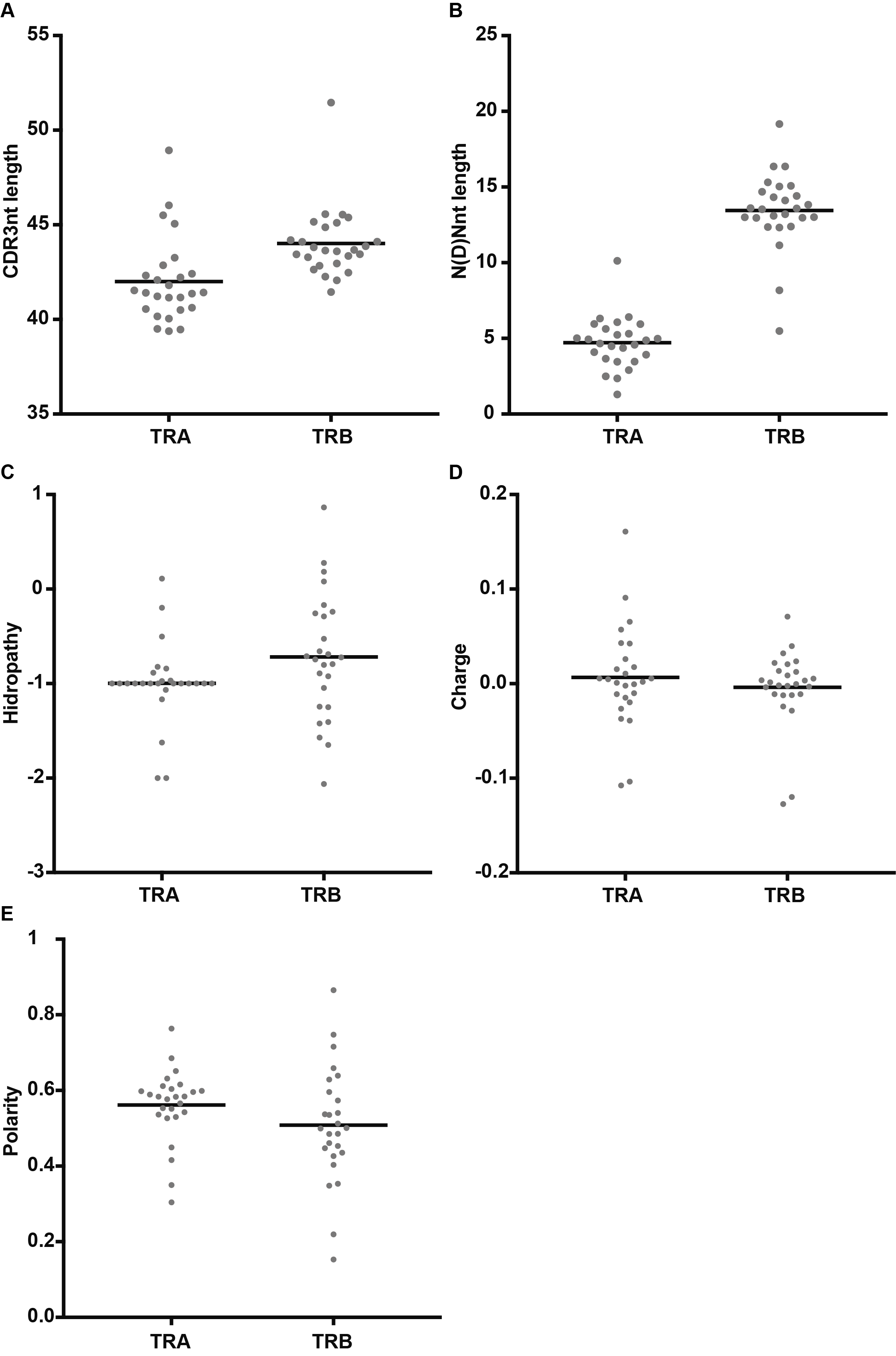
**Supplementary Figure 7. CDR3nt length and N(D)Nnt length and biochemical properties of the central region (5aa) of CDR3 TRA and TRB sequences in TILs from initial cultures.** (A) The mean of CDR3 nt length was 42 nt and 44 nt for TRA and TRB sequences, respectively. (B) The mean of N(D)N nt length was 5 nt and 13nt in TRA and TRB sequences, respectively. (C) Hydrophobicity values obtained in different samples. The mean values were -0.99 and 0.72 for TRA and TRB sequences, respectively. (D) Charge values obtained in different samples. The mean values were around 0 for both TRA and TRB sequence groups. (E) Polarity values of different samples. The mean values were 0.56 and 0.51 for TRA and TRB sequences, respectively.

**Supplementary Figure 8. TCR repertoire diversity analysis in TIL from initial cultures.** (A) Correlation of nS-W diversity indices between TRA and TRB sequences in different sections of analyzed biopsies. A significant correlation was observed between both sequence groups (p < 0.0001). (B) Dispersion and mean of nS-W diversity indices obtained in TRA and TRB sequences.


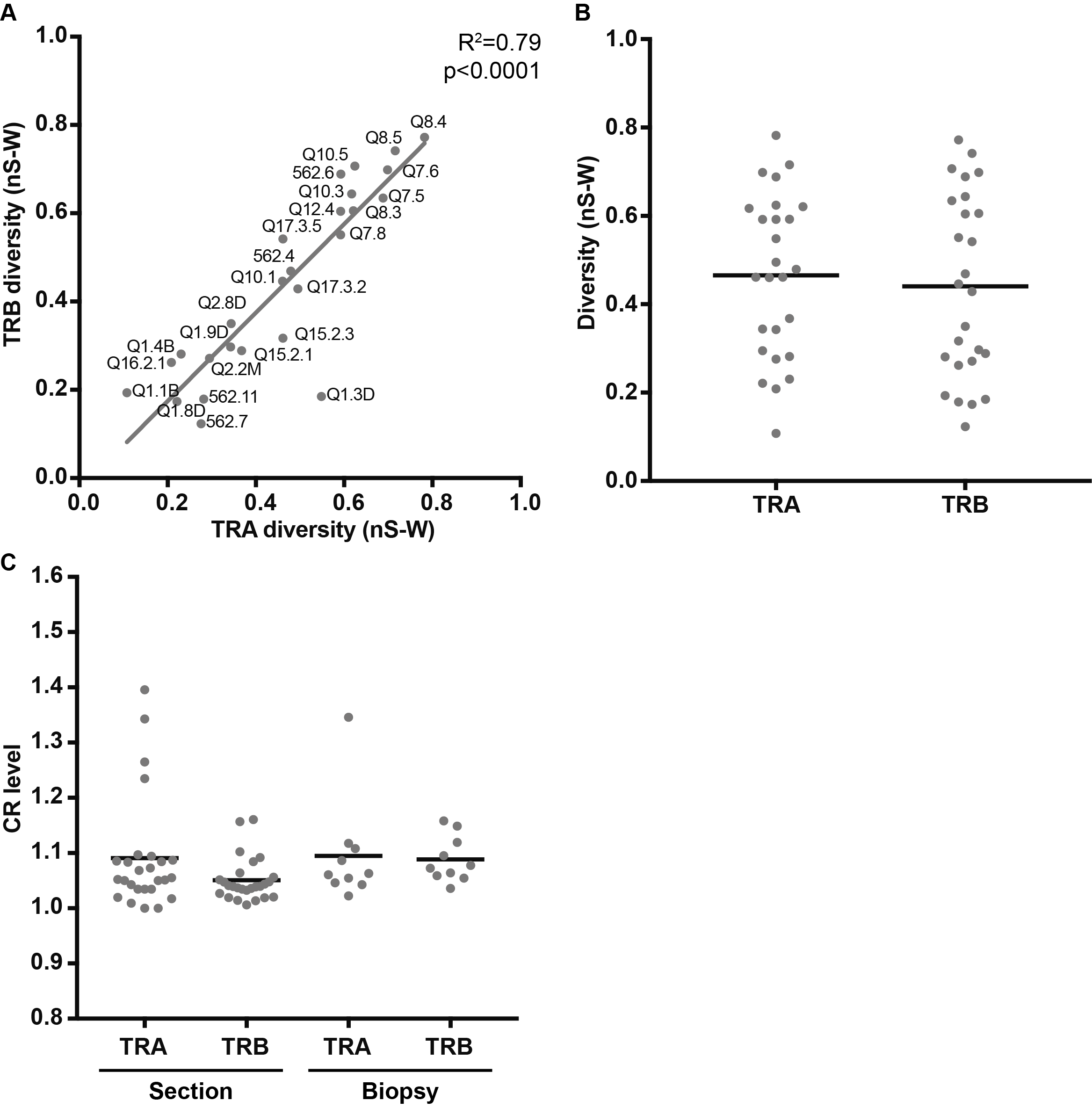

Supplement: Supplementary file 2 [file SupplementaryFile1.docx]
